# Soundscapes as Sonic Seasoning of Chocolate: Effects on Taste Perception, Affect, and Liking

**DOI:** 10.3390/foods15122142

**Published:** 2026-06-13

**Authors:** Marcos Eduardo Valdés-Alarcón, Andrea Cristina Aulestia-Vizcaíno, Alexander Sánchez-Rodríguez, Rodobaldo Martínez-Vivar, Gelmar García-Vidal, Reyner Pérez-Campdesuñer

**Affiliations:** 1 Tourism System Research Group (GIST), Faculty of Gastronomic Sciences and Tourism, Universidad UTE, Quito 170512, Ecuador; marcos.valdes@ute.edu.ec (M.E.V.-A.); andreac.aulestia@ute.edu.ec (A.C.A.-V.); 2 Business Administration Research Group (GADEP), Faculty of Engineering Sciences and Industries, Universidad UTE, Quito 170512, Ecuador; 3 Business Administration Research Group (GADEP), Faculty of Law, Administrative and Social Sciences, Universidad UTE, Quito 170512, Ecuador; rodobaldo.martinez@ute.edu.ec (R.M.-V.); gelmar.garcia@ute.edu.ec (G.G.-V.); reyner.perez@ute.edu.ec (R.P.-C.)

**Keywords:** soundscape, chocolate, taste perception, sensory evaluation, crossmodal correspondences, valence, arousal, sonic seasoning

## Abstract

This study examines how auditory contexts, or soundscapes, shape chocolate taste perception, affective response, hedonic liking, and the extent to which emotion mediates these effects. Using a within-subjects design with 120 participants aged 18–25 years, four auditory conditions were compared: silence, natural soundscape, relatively low-pitched soundscape, and relatively high-pitched soundscape. Participants evaluated perceived bitterness, sweetness, acidity, emotional valence, arousal, and overall liking after tasting the same 65% dark chocolate under each auditory condition. The results showed that auditory context significantly modulated taste perception, affective response, and liking. The natural soundscape produced the most favorable profile, increasing liking and emotional valence while reducing arousal. In contrast, the relatively high-pitched condition increased arousal and enhanced perceived acidity (Δ ≈ 6.77 VAS points). Effect sizes indicated stronger effects on arousal (partial η^2^ ≈ 0.46), liking (partial η^2^ ≈ 0.29), acidity (partial η^2^ ≈ 0.28), and valence (partial η^2^ ≈ 0.26) than on sweetness perception (partial η^2^ ≈ 0.05). Mediation analysis showed that emotional valence partially explained the relationship between the natural soundscape and liking, whereas arousal did not play a significant mediating role. These findings suggest that auditory environments influence chocolate evaluation through both affective and crossmodal pathways. Overall, the study provides controlled evidence that sound can function as a relevant contextual variable in multisensory chocolate-tasting experiences, with implications for sensory evaluation, gastronomy, and experience design.

## 1. Introduction

Flavor perception and hedonic evaluation are increasingly understood not as purely physiological outcomes, but as products of a broader multisensory integration process in which environmental cues interact with gustatory inputs to shape perceived taste intensity and consumers’ emotional responses. In gastronomy, eating is rarely a decontextualized act; rather, it unfolds within staged experiences where atmosphere, service, and sensory framing influence how a product is interpreted and valued. Accordingly, a growing body of research shows that gustatory experience depends not only on the physicochemical properties of foods, but is systematically modulated by visual, olfactory, tactile, and auditory information integrated within the human perceptual system [1,2].

Within this multisensory framework, crossmodal correspondences constitute a key mechanism explaining how stimuli in one sensory modality can influence perception in another without a direct physical relationship. These associations reflect a combination of perceptual constraints, learned regularities, and cultural conventions, and have been shown to shape judgments of taste intensity, emotional response, and overall product evaluation [2,3]. More specifically, auditory–gustatory correspondences show that sound properties can be systematically associated with basic taste qualities, including sweetness, bitterness, sourness/acidity, and other flavor-related impressions [4,5]. In this context, sound has emerged as a relevant cue in food perception, as auditory properties—such as pitch, frequency, timbre, musical structure, and broader soundscapes—can bias sensory attributes, emotional response, and hedonic evaluation [6,7,8,9,10,11].

Within this field, research on sonic seasoning has shown that sound can systematically influence taste and flavor-related judgments. The foundational study by Crisinel et al. [6] demonstrated that modifying the sonic properties of a background soundtrack could alter the perceived taste of food, including in a chocolate-related tasting context. Subsequent chocolate-related research further showed that music can influence multisensory tasting evaluations, supporting the idea that auditory cues may alter taste-related judgments and broader product appraisal during tasting [12]. In particular, high-pitched sounds have often been associated with sweetness or sourness/acidity, whereas lower-pitched sounds have been more frequently associated with bitterness or heavier sensory impressions [4,5]. However, these associations should not be interpreted as universal or purely perceptual, because auditory influences on tasting may also depend on psychoacoustic properties, musical structure, affective responses, familiarity, and congruency [7,8,9,10].

More recent chocolate-focused studies have attempted to disentangle the perceptual and emotional components of sonic seasoning. Reinoso-Carvalho et al. [7] compared music selected to induce specific emotional responses with music selected for crossmodal congruency with sensory attributes of the tasting experience, showing that emotional routes may sometimes produce stronger and more globally meaningful effects than purely crossmodal strategies. In a related chocolate study, Reinoso-Carvalho et al. [8] further demonstrated that music can trigger both crossmodal and emotional effects in multisensory tasting experiences, influencing variables such as texture perception, flavor intensity, emotional response, and purchase-related evaluations. These studies are especially relevant because they show that sonic seasoning should not be reduced to pitch–taste matching alone; rather, its effects may emerge from the interaction between crossmodal correspondences and affective mechanisms.

Soundscapes—understood as coherent acoustic environments—are also relevant in this context because they can elicit affective states such as calmness, pleasantness, or activation, thereby influencing attention, expectations, and sensory interpretation [13]. Natural soundscapes, such as water, wind, or birdsong, are frequently associated with positive affective states, relaxation, and enhanced experiential quality, whereas more activating auditory stimuli may increase arousal and modify the perception of specific taste attributes [11,12,13]. From this perspective, emotion can function as a mediating mechanism linking auditory context to hedonic evaluation. This is particularly relevant when using standardized affective measures such as the Self-Assessment Manikin (SAM), which captures core dimensions of emotional valence and arousal.

Despite the sustained growth of this research stream, much of the empirical evidence has been generated in a limited set of geographic and product contexts, often using standardized foods or beverages with relatively limited territorial anchoring. This constrains the generalizability of current knowledge and leaves open relevant questions about how crossmodal and affective dynamics unfold in emblematic foods from producing regions in the Global South. In particular, there remains a visible gap in studies that jointly integrate cross-modal auditory manipulation, emotional responses, hedonic evaluations, and mediation analyses in products with strong origin-based identity—precisely the type of evidence that can inform gastronomic storytelling and experience design around terroir-linked foods.

Chocolate provides an appropriate product context for addressing this question. It is a product with a complex sensory profile in which bitterness, sweetness, acidity, texture, and aromatic notes interact in nuanced ways, and its appreciation is strongly shaped by contextual and emotional factors [14,15]. Chocolate is also widely used in sensory research because it reliably elicits measurable hedonic responses, making it well-suited for controlled experimental testing of multisensory influences [13,14,15]. In the present study, a 65% dark chocolate sample from Ecuador was used as the tasting stimulus. Ecuador is internationally recognized as a major producer of fine-flavor cacao [16]; however, the origin of the chocolate is treated here as part of the empirical product context rather than as an explanatory mechanism. The study does not test country-of-origin effects, terroir perception, authenticity, or quality expectations. Instead, it uses this chocolate stimulus to examine how auditory environments influence taste perception, affective response, and hedonic evaluation under controlled tasting conditions.

Against this background, the present study evaluates how different auditory environments—relatively low-pitched soundscape, relatively high-pitched soundscape, natural soundscape, and silence—modify perceived taste attributes, emotional responses, and hedonic liking during chocolate tasting. In addition, the study examines whether emotional response mediates the relationship between auditory context and liking. The contribution of the study does not lie in claiming, for the first time, that sound can influence chocolate tasting, nor in attributing the observed effects to the Ecuadorian origin of the product. Rather, it lies in integrating taste-intensity perception, emotional valence, arousal, hedonic liking, and mediation analysis within a single controlled within-subjects design. This allows us to examine whether auditory environments influence chocolate evaluation through sensory crossmodal correspondences, affective pathways, or both.

Based on this framework, the following hypotheses are proposed: H1. Auditory environment produces significant changes in perceived taste-intensity attributes and overall liking during chocolate tasting. H2. Specific auditory environments produce directional effects on emotional response and perceived sensory attributes during chocolate tasting. H2a. Natural soundscapes increase liking and promote a calmer emotional response, reflected in higher emotional valence and/or lower arousal. H2b. Relatively high-pitched sounds increase emotional activation, reflected in higher arousal, and increase perceived acidity. H3. Emotion mediates the effect of auditory environment on liking during chocolate tasting.

To test these hypotheses, the study uses a controlled tasting design that manipulates auditory environment and assesses three outcomes: perceived taste intensity, core affect (valence and arousal), and overall liking. This design enables the evaluation of both direct crossmodal effects and affective contributions to chocolate appraisal.

## 2. Materials and Methods

### 2.1. Study Design, Participants, and Sample Size

This study used a controlled within-subjects (repeated-measures) design to examine how auditory environments modulate sensory perception, emotional response, and overall liking during chocolate tasting. Each participant completed all four auditory conditions (relatively low-pitched, relatively high-pitched, natural soundscape, and silence) in a single session to reduce interindividual variability. The order of conditions was counterbalanced using a 4 × 4 Latin square (Section 2.3).

Participants were adult chocolate consumers recruited through non-probabilistic convenience sampling in a university setting. The final sample comprised 120 participants aged 18–25 years, including 66 women and 54 men. Eligibility criteria included regular chocolate tasting, self-reported normal hearing, and absence of cacao allergies or intolerances. Individuals reporting taste or smell disorders, hearing impairments, or recent consumption of substances that could interfere with gustatory perception were excluded.

The target sample size was set at 120 participants. This number was considered adequate for a repeated-measures design with four auditory conditions because each participant provided observations under all experimental conditions, increasing sensitivity to within-person differences. The sample size was intended to provide sufficient power to detect medium-sized within-subject effects in the primary repeated-measures ANOVA and to allow stable estimation of indirect effects in the exploratory mediation analysis using bootstrap confidence intervals. This decision was guided by general recommendations for sample-size justification in behavioral research and by methodological work on mediation analysis, which emphasizes the need for larger samples when estimating indirect effects [17,18,19]. All participants completed the experiment and were included in the analyses.

### 2.2. Stimuli and Apparatus

**Chocolate stimulus:** The stimulus was dark chocolate (65% cacao). Each participant received a 5 g portion per condition from the same product lot, served in identical containers with randomized identifiers. Samples were stored under controlled conditions and used within 12 h of opening. Participants consumed each portion within 60 s and completed the evaluations immediately afterward.

**Auditory stimuli:** Four auditory conditions were used: a relatively low-pitched musical soundscape, a relatively high-pitched musical soundscape, a natural soundscape, and silence. The auditory stimuli were prepared as separate 60-s stereo WAV files and standardized at 48 kHz and 24-bit resolution. The relatively low-pitched and relatively high-pitched conditions were complex musical soundscapes rather than pure tones; therefore, these labels refer to their comparative acoustic profiles within the stimulus set rather than to fixed frequency-band categories. The natural soundscape consisted of environmental sounds, including birds, wind, and water, whereas the silence condition was represented by a 60-s digital silence WAV file.

To improve transparency and reproducibility, the four auditory stimuli are provided as Supplementary audio files with the revised submission. Each file is accompanied by technical metadata and an acoustic audit reporting duration, file format, sampling rate, bit depth, channel configuration, RMS level, approximate integrated loudness, peak level, clipping verification, dominant frequency, spectral centroid, spectral bandwidth, band-energy distribution, temporal RMS variability, source/license information, and SHA-256 hash values. These materials allow the auditory stimuli to be inspected and independently evaluated by readers.

Because the original audio files used during data collection were not formally archived in a public repository at the time of the experiment, the files supplied with the revised manuscript are reported as reconstructed and standardized auditory stimuli. They were prepared in accordance with the experimental criteria described in the protocol, including auditory condition, duration, technical format, stereo configuration, intensity control, and absence of clipping. These files are provided to support transparency and reproducibility of the auditory conditions, but they should not be interpreted as fully documented archival copies of the exact original playback files.

### 2.3. Procedure and Measures

Sessions were conducted in a controlled environment. After standardized instructions and a brief sociodemographic questionnaire, participants completed four tasting trials (one per auditory condition) in a single session. Condition order was counterbalanced using a 4 × 4 Latin square (Table 1), with participants randomly assigned to one of four sequences (*n* = 30 per sequence).

In each trial, participants were exposed to the assigned auditory environment, consumed a 5 g portion of chocolate, and immediately recorded their evaluations. To minimize carryover effects, participants rinsed with water, consumed a neutral cracker, and waited at least 60 s before proceeding to the next trial. The chocolate stimulus, portion size, and environmental conditions were kept constant; only auditory context varied.

Participants were not explicitly informed that all chocolate samples came from the same product lot. Samples were presented in identical containers with randomized identifiers to reduce expectancy effects and avoid drawing attention to the sameness of the chocolate across conditions. However, because participants tasted four portions of the same chocolate in a single session, some may have inferred the samples were identical. This possibility is acknowledged as a limitation of the study.

Measures: The independent variable was auditory environment (within-subjects: four levels). The dependent variables were: (i) perceived intensity of bitterness, sweetness, and acidity measured using 0–100 visual analog scales; (ii) overall liking measured on a 9-point hedonic scale; and (iii) emotional response measured using the Self-Assessment Manikin (SAM), capturing valence and arousal.

### 2.4. Statistical Analysis

Analyses began with descriptive statistics and diagnostic checks, including assessment of distributional behavior and repeated-measures assumptions. Hypotheses H1, H2a, and H2b were tested using repeated-measures ANOVA with auditory environment as the within-subject factor (four levels). Effect sizes were reported as partial η^2^.

H1 was evaluated through the overall effect of auditory environment on taste intensity, liking, and emotional responses. H2a and H2b were tested using planned contrasts comparing specific auditory conditions (natural soundscape and relatively high-pitched sound, respectively) against the remaining conditions. To control for Type I error, *p*-values for planned comparisons were adjusted using the Holm procedure. The significance level was set at α = 0.05.

Mediation analysis (H3): Mediation was examined using a parallel mediation model in which auditory environment was the predictor, valence and arousal were specified as mediators, and liking was the outcome. Because each participant contributed repeated observations across the four auditory conditions, the mediation analysis was specified using within-participant contrasts rather than treating all condition-level observations as fully independent. Silence was used as the reference condition, and three contrast variables were computed for each participant to represent the intra-individual differences between silence and each auditory condition: natural soundscape, relatively low-pitched sound, and relatively high-pitched sound. This contrast-based specification reduces the risk of pseudoreplication by focusing on within-person changes attributable to auditory context rather than on between-person differences.

Indirect effects were estimated using bootstrap resampling of the product of coefficients (a·b), and statistical significance was assessed using bias-corrected bootstrap confidence intervals. All analyses were conducted in R version 4.5.3 using RStudio version 2026.01.2, and the mediation model was estimated using the mediation package version 4.5.1. Although the use of within-participant contrasts partially accounts for the repeated-measures structure, the model does not constitute a full multilevel mediation analysis. While a multilevel mediation framework would provide a more rigorous modeling of the nested structure of repeated observations, the present approach offers a parsimonious first approximation of within-participant indirect effects and is appropriate for the exploratory scope of the study. Therefore, the mediation findings are interpreted cautiously, and future research should use multilevel mediation or multilevel structural equation modeling to explicitly model subject-level clustering and separate within- and between-participant effects [20,21].

### 2.5. Ethical Considerations

This research involved a minimal-risk, non-clinical sensory evaluation in which adult participants (18–25 years) tasted a commercially available chocolate sample under controlled auditory conditions and completed non-invasive self-report measures (VAS, hedonic liking, and SAM). Participation was voluntary, and written informed consent was obtained prior to data collection. Participants were informed about the study procedure, the absence of penalties for non-participation, and their right to discontinue at any time without consequences. Screening procedures were implemented to reduce foreseeable risks associated with ingestion (e.g., excluding individuals reporting cacao allergy or intolerance) and to protect participants’ welfare throughout the session.

The study was conducted in accordance with the Ecuadorian regulatory framework governing CEISH/CEAS ethics oversight (Ministry of Public Health), established through Acuerdo Ministerial No. 4889 and reaffirmed/updated via Acuerdo Ministerial No. 00005-2022 (Registro Oficial Quinto Suplemento No. 118, 2 August 2022). These regulations emphasize the protection of the dignity, rights, safety, and well-being of human participants in research protocols, consistent with the stated purpose of CEISH committees. In line with this framework, data were collected and managed using anonymous identifiers, and no biometric data, clinical measures, or personally identifying records were collected; results are reported in aggregate form. Finally, all procedures adhered to internationally recognized ethical principles for research involving humans, including the Declaration of Helsinki (2013 revision), particularly regarding informed consent, privacy, and the minimization of foreseeable risks and burdens.

The experimental protocol was designed to mirror realistic contexts of chocolate tasting, in which sound is a natural part of the dining atmosphere, such as cafés, chocolateries, and guided tastings. The natural soundscape condition reflects calm, immersive environments commonly used to enhance food experiences, whereas the relatively low- and high-pitched conditions represent tonal sound profiles that may be intentionally created through curated music, sound design, or controlled playback in tasting environments. By keeping the chocolate stimulus constant and manipulating only the auditory environment under standardized tasting and palate-cleansing procedures, the study isolates the contribution of sound to perceived taste intensity, affective responses (valence and arousal), and overall liking, providing evidence to inform multisensory experience design in gastronomic settings.

## 3. Results

### 3.1. Data Integrity and Assumption Checks

The dataset was complete, with one observation per participant per auditory condition and no missing values. Sphericity was assessed using Mauchly’s test. The assumption held for bitterness, acidity, liking, valence, and arousal (*p* > 0.05). For sweetness, sphericity was violated (χ^2^(5) = 15.00, *p* = 0.010); therefore, results are reported using the Greenhouse–Geisser correction (ε = 0.92), with comparable results under Huynh–Feldt correction.

Residual diagnostics indicated minor deviations from normality for acidity and arousal (*p* < 0.05), whereas the other variables met the normality assumption. Given the sample size and the bounded nature of the scales, these deviations were considered non-critical. Outlier analysis based on standardized residuals (|z| > 3) showed low proportions (0.0–0.63%). A sensitivity analysis excluding these observations produced negligible changes in means (Δ < 0.33 VAS points; Δ < 0.03 in liking), and all data were retained.

### 3.2. Descriptive Results by Auditory Condition

Table 2 presents the means and standard deviations for sensory intensity, liking, and emotional response across conditions.

Descriptive patterns indicate systematic variation across auditory conditions. Bitterness was highest under the relatively low-pitched condition and lowest under the natural soundscape. Sweetness showed minimal variation across conditions. Acidity was highest under the relatively high-pitched condition and lower under silence and nature.

Liking was highest under the natural soundscape and lowest under the relatively high-pitched condition. Emotional responses followed a similar pattern: valence was highest under the natural soundscape, while arousal was highest under the relatively high-pitched condition and lowest under nature.

### 3.3. Inferential Results: Repeated-Measures ANOVA

The repeated-measures ANOVA revealed a statistically significant effect of auditory environment on all dependent variables (Table 3). Auditory environment significantly affected bitterness (partial η^2^ = 0.223), sweetness (partial η^2^ = 0.050), and acidity (partial η^2^ = 0.279). Effects were moderate for bitterness and acidity and small for sweetness. Significant effects were also observed for liking (partial η^2^ = 0.288), valence (partial η^2^ = 0.263), and arousal (partial η^2^ = 0.455), indicating moderate-to-large effect sizes, particularly for arousal.

Notably, although the effect on perceived sweetness reached statistical significance, its magnitude was comparatively small relative to the other outcomes. Therefore, the sweetness result should be interpreted as a modest auditory-related shift or bias in ratings under the present experimental conditions, rather than as evidence that sweetness is inherently less sensitive to auditory variation.

### 3.4. Planned Contrasts (H2a–H2b)

Planned contrasts were conducted to test directional hypotheses (Table 4).

The natural soundscape significantly increased liking (Δ = 0.824) and valence (Δ = 0.839), and decreased arousal (Δ = −0.973) relative to the mean of the other conditions (all *p* < 0.001). The relatively high-pitched condition significantly increased arousal (Δ = 1.031) and perceived acidity (Δ = 6.769 VAS points) compared with the other conditions (all *p* < 0.001).

**Figure 1:**
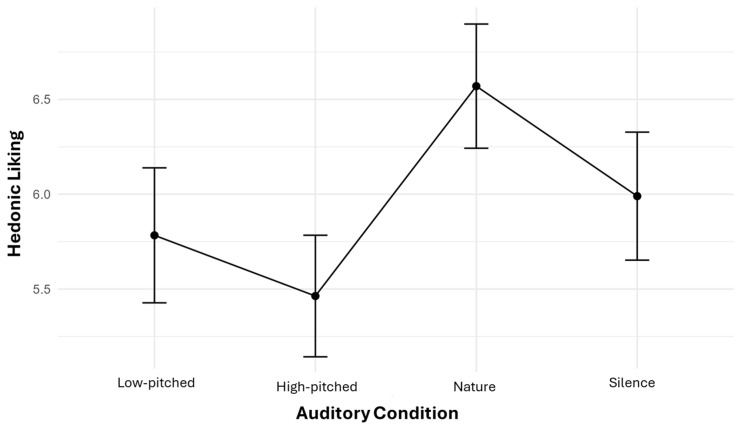
Hedonic liking by auditory condition. Error bars represent 95% confidence intervals around the adjusted marginal means.

**Figure 2:**
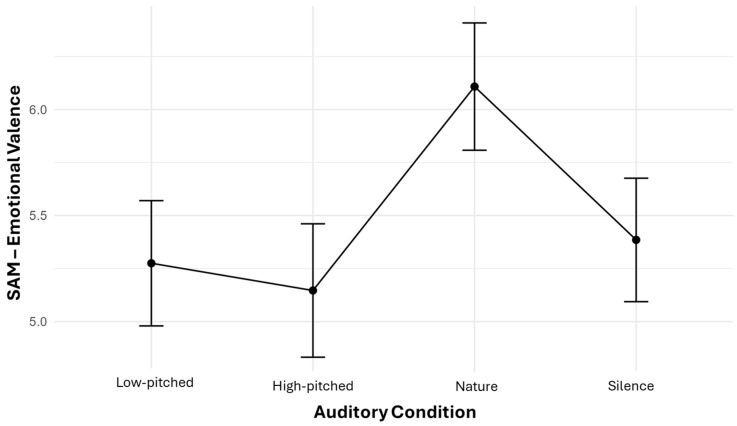
Emotional response (SAM) by auditory condition: emotional valence. Error bars represent 95% confidence intervals around the adjusted marginal means.

**Figure 3:**
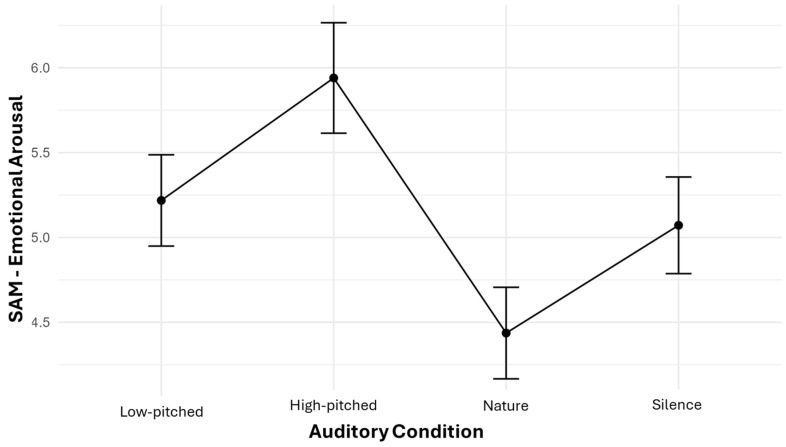
Emotional response (SAM) by auditory condition: emotional arousal. Error bars represent 95% confidence intervals around the adjusted marginal means.

Figure 1, Figure 2 and Figure 3 present the adjusted marginal means for liking, valence, and arousal across auditory conditions.

### 3.5. Mediation Analysis (H3)

A mediation model was estimated to assess whether emotional response mediates the relationship between auditory environment and liking (Table 5). To reduce pseudoreplication concerns associated with the within-subjects design, the analysis was based on within-participant contrasts between silence and each auditory condition, rather than on treating all repeated condition-level observations as independent. This contrast-based approach focuses on intra-individual differences attributable to auditory context and provides a parsimonious first approximation of within-participant indirect effects. Nevertheless, because the model did not explicitly estimate subject-level random effects, the mediation results should be interpreted as exploratory and with appropriate caution.

The natural soundscape significantly increased emotional valence (β = 0.2537, *p* < 0.001), and valence was positively associated with liking (β = 0.1634, *p* = 0.009). The indirect effect through valence was statistically significant (β = 0.0415, *p* = 0.045), indicating partial mediation.

The direct effect of the natural soundscape on liking remained significant (β = 0.1658, *p* = 0.036), as did the total effect (β = 0.1825, *p* = 0.015). For the relatively high-pitched condition, a significant negative direct effect on liking was observed (β = −0.1860, *p* = 0.013).

**Figure 4:**
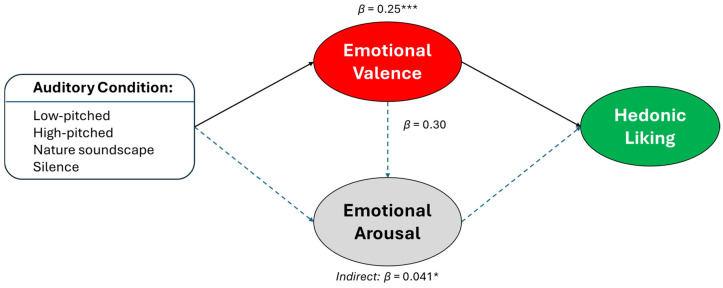
Mediation model of the effect of auditory environment on hedonic liking through emotional response. Values shown on the arrows are standardized coefficients (β). Asterisks next to the coefficients indicate the statistical significance of the corresponding path coefficient: * *p* < 0.05; *** *p* < 0.001. The indirect effect through emotional valence was estimated using bootstrap confidence intervals.

Figure 4 summarizes the mediation model.

Overall, the results show that the auditory environment significantly influences sensory perception, emotional response, and hedonic liking of chocolate. The observed effects are consistent with the proposed hypotheses: the natural soundscape is associated with higher liking and valence and lower arousal, whereas the relatively high-pitched condition is associated with increased arousal and perceived acidity. In addition, the mediation analysis indicates that emotional valence helps explain the relationship between auditory context and liking.

## 4. Discussion

Taken together, the repeated-measures ANOVA, planned contrasts, and mediation analysis reveal a consistent pattern in which auditory context systematically shapes both the sensory profile of chocolate and the associated affective and hedonic responses. Sound influenced perceived bitterness, acidity, sweetness, liking, and core affect, although the magnitude of these effects differed across outcomes. The strongest effects were observed for arousal, liking, acidity, and emotional valence, whereas the effect on sweetness was statistically significant but comparatively small.

The sensory results suggest that auditory context does not uniformly modify all taste attributes. Instead, different auditory profiles appear to emphasize specific dimensions of the tasting experience. The relatively high-pitched condition produced a clear increase in perceived acidity, indicating that brighter or more activating auditory environments may accentuate sharper sensory notes. This pattern is consistent with auditory–gustatory correspondence research linking higher-pitched sounds with sourness/acidity-related associations. In contrast, the relatively low-pitched condition was associated with higher perceived bitterness, suggesting a possible correspondence between lower-pitched auditory profiles and heavier or more bitter taste impressions.

The significant but small effect observed for sweetness also deserves consideration. Although sweetness ratings varied less across auditory conditions than bitterness, acidity, liking, valence, or arousal, the result suggests that auditory context may produce modest shifts in sweetness ratings. This finding is consistent with the broader sonic seasoning literature, in which sweetness has often been linked to higher-pitched or positively valenced auditory stimuli. However, in the present study, the effect size for sweetness was comparatively small, indicating that sweetness was not the primary taste attribute modulated by the auditory manipulation. Therefore, the sweetness effect should be interpreted as a modest auditory-related bias in perceived sweetness under the present experimental conditions, rather than as a strong or practically dominant effect.

Beyond taste perception, the findings also show that auditory context shaped the affective and hedonic dimensions of chocolate tasting. The natural soundscape produced the most favorable profile, increasing liking and emotional valence while reducing arousal. This pattern suggests that calm and positively valenced auditory environments may enhance chocolate evaluation primarily by improving the affective quality of the tasting experience. Conversely, although the relatively high-pitched condition increased arousal, it did not enhance liking, indicating that activation alone is not necessarily beneficial for hedonic appraisal.

Importantly, the mediation analysis helps clarify the mechanism underlying these effects. The natural soundscape increased emotional valence, which in turn was positively associated with liking, resulting in a significant indirect effect consistent with partial mediation. In contrast, although relatively high-pitched sounds increased arousal, this dimension did not contribute to explaining liking. These findings identify emotional valence—rather than arousal—as the primary affective pathway linking auditory context to consumer response. This interpretation is consistent with recent evidence showing that emotional valence, more than arousal, can influence taste perception, including perceived sweetness and bitterness [22].

At the same time, the persistence of a direct effect of the natural soundscape on liking suggests that valence alone does not fully account for the observed relationship. Additional mechanisms beyond emotion, such as crossmodal congruency, attentional modulation, or context-induced expectations, may also contribute to the observed effects and should be examined more directly in future research. Thus, the overall pattern supports a dual-route interpretation in which auditory environments influence chocolate tasting through both sensory crossmodal correspondences and affective pathways.

### 4.1. Auditory Atmospherics as Crossmodal and Affective Drivers of Chocolate Experience

The findings confirm that the auditory environment acts as a relevant modulator of the tasting experience, influencing sensory perception, hedonic evaluation, and emotional response in an integrated manner. This convergence is consistent with the crossmodal interaction and sonic seasoning literature, which demonstrates that auditory stimuli can alter taste perception and food evaluation through their integration with sensory processing [11,12,23]. The smaller but statistically significant sweetness effect further suggests that auditory context may also bias sweeter taste impressions, although this pathway was weaker than the acidity and bitterness effects observed in the present study.

These findings should be interpreted in relation to prior chocolate-based sonic seasoning studies. Crisinel et al. [6] provided early evidence that changing the sonic properties of a background soundtrack can systematically modulate the taste of food, including chocolate-related tasting. Later, Reinoso-Carvalho et al. [7] advanced this literature by distinguishing between music designed to operate through emotional induction and music designed to operate through crossmodal congruency with sensory attributes of the tasting experience. Their findings, together with those of Reinoso-Carvalho et al. [8], showed that sonic seasoning effects may depend not only on auditory–gustatory matching, but also on the emotional states elicited by the auditory stimulus [24].

The present study is consistent with this dual-route interpretation but extends it in three ways. First, it examines Ecuadorian chocolate, an origin-linked product from a Latin American cacao-producing context that has received limited attention in sonic seasoning research. Second, it simultaneously evaluates taste-intensity attributes, emotional valence, arousal, and hedonic liking. Third, it formally tests whether emotional response mediates the effect of auditory context on liking. In this sense, the study does not merely confirm that sound can bias taste-related judgments; rather, it helps clarify how perceptual and affective mechanisms jointly contribute to chocolate evaluation.

Specifically, the results support a dual-process interpretation in which auditory context influences food evaluation through: (i) crossmodal correspondences that bias the perception of specific sensory attributes, and (ii) affective mechanisms that shape emotional state and, indirectly, hedonic judgment. The increase in perceived acidity under the relatively high-pitched condition is consistent with auditory–gustatory correspondence effects, particularly prior evidence linking higher-pitched sounds with sourness/acidity-related associations [4,5]. In contrast, the positive effect of the natural soundscape on liking appears to operate partly through emotional valence. This distinction is important because it shows that different auditory profiles may influence chocolate experience through different mechanisms.

Within this framework, identifying emotional valence as a partial mediator provides a more precise explanation of how auditory context translates into liking. A more positive affective state contributes to more favorable evaluations, but it does not fully account for the observed effects, indicating that perceptual and attentional mechanisms may also play a role [11,23]. This finding refines prior work by empirically separating the contributions of affect and crossmodal perceptual bias.

The observed pattern of arousal further supports this interpretation. Although relatively high-pitched sounds increased activation levels, this dimension did not translate into higher liking, suggesting that arousal alone is not a sufficient driver of hedonic evaluation. This result nuances previous assumptions that more stimulating environments necessarily enhance the tasting experience, instead highlighting the importance of affective quality, particularly valence, over activation intensity in shaping consumer responses [25].

Overall, these results extend the crossmodal and multisensory literature by integrating sensory perception, emotional response, and hedonic evaluation within a single analytical framework and by providing empirical evidence from an origin-linked product in an emerging-economy context. This contribution helps bridge the gap between controlled experimental research and applied gastronomic experience design.

### 4.2. Managerial Implications

The findings indicate that the auditory environment is not a neutral background element, but an active driver of the sensory, affective, and hedonic dimensions of consumer experience. Therefore, sound may be managed as a strategic servicescape variable rather than treated as incidental [26,27]. Within the empirical scope of this study, this involves standardizing playlists or soundscapes according to specific experience goals, particularly in contexts such as guided tastings, sensory evaluation sessions, chocolateries, or experience-oriented gastronomic events, thereby reducing uncontrolled variability in sensory perception and evaluation [9,11].

A key implication concerns the role of emotional valence. Since the natural soundscape increased both valence and liking, designing auditory environments that evoke calmness and positive affect may be particularly appropriate for chocolate experiences oriented toward enjoyment, relaxation, and favorable product appraisal. Aligning sound design with the product’s origin narrative—such as cacao, biodiversity, and terroir—may also help reinforce experiential coherence and perceived authenticity, in line with crossmodal correspondences and sonic seasoning principles [11,28]. However, this implication should be interpreted cautiously, as the present study did not directly measure perceived authenticity, origin associations, or place-based meanings.

At a more operational level, auditory strategies can be tailored to the product’s sensory profile and the intended experience. Calmer soundscapes may be more appropriate for products positioned around smoothness or hedonic enjoyment, whereas more activating auditory environments may emphasize intensity-related attributes, such as roasted or acidic notes. However, the results suggest that increased activation does not necessarily translate into higher liking, highlighting the need to balance sensory enhancement with affective impact [13].

For guided tastings and experiential events, implementation should follow standardized protocols, including volume levels, reproduction mode (e.g., speakers vs. headphones), exposure duration, and sequencing within the tasting process. Such standardization reduces operational variability and enhances the consistency and replicability of the experience. Additionally, digital tools such as QR codes linking to curated playlists may be explored as low-cost tools to extend the designed auditory experience beyond the point of tasting, particularly for premium or experiential products. Nevertheless, these applications should be understood as design implications derived from the observed effects on sensory perception, emotional response, and liking, rather than as directly tested outcomes.

Finally, managerial decisions should be supported by systematic measurement [28]. Future field-based A/B testing of alternative soundscapes could be conducted in realistic tasting or consumption contexts, such as guided tastings, chocolateries, cafés, hospitality settings, product demonstrations, or controlled home-use studies. Such studies would help determine whether the sensory, affective, and hedonic effects observed in this controlled experiment translate into behavioral or business-related outcomes, such as willingness to pay, choice behavior, repeat purchase, dwell time, customer satisfaction, or liking ratings. Where feasible, these approaches could be complemented with non-invasive, real-time measurement techniques, allowing firms to integrate behavioral and perceptual data in the design and optimization of multisensory experiences [29,30].

### 4.3. Limitations and Recommendations for Future Research

This study presents several limitations that should be considered when interpreting the findings and that open avenues for future research. First, the use of university-based convenience sampling and the restriction of the sample to young adults aged 18–25 years constitute key limitations that directly constrain the generalizability of the findings. Although this sampling strategy was appropriate for a controlled within-subjects design aimed at reducing extraneous variability and increasing sensitivity to crossmodal effects, the results should not be interpreted as representative of the broader population of chocolate consumers. Instead, they should be understood as evidence of sensory, affective, and hedonic responses within a specific group of young adult university participants. Future research should replicate the study using more heterogeneous samples across age, socioeconomic background, cultural context, tasting habits, and familiarity with Ecuadorian chocolate. Such extensions would help determine whether the observed auditory effects are stable across consumer segments or moderated by demographic and experiential factors [11].

Second, although the controlled experimental setting strengthens internal validity, it reduces ecological validity relative to real tasting contexts, where multiple sensory, spatial, and social cues interact simultaneously [31,32]. Real-life chocolate tasting often takes place in cafés, homes, festivals, chocolateries, or guided tasting environments, where ambient noise, interpersonal interaction, and contextual atmosphere may shape the tasting experience in ways not captured under laboratory conditions. Accordingly, the present findings should not be assumed to transfer directly to naturalistic gastronomic settings. Future research should incorporate field-based and ecologically embedded designs to assess the robustness of auditory effects under more realistic multisensory conditions. In addition, future studies should compare different sound delivery modalities, such as speakers versus headphones, and examine how playback conditions influence perceptual, affective, and hedonic responses.

Third, the auditory stimuli used in this study were designed to represent clearly distinguishable relatively low-pitched, relatively high-pitched, and natural-soundscape profiles, but the original playback files were not formally archived in a public repository at the time of data collection. This limits full independent replication of the exact auditory conditions used in the original experiment. In addition, the stimuli were not subjected to prior perceptual validation; therefore, the terms “relatively low-pitched” and “relatively high-pitched” should be interpreted as referring to the intended dominant auditory profile of each soundscape rather than to precisely delimited frequency bands. Similarly, the “natural” condition should be understood as a specific composite soundscape combining water, wind, and birdsong, rather than as a homogeneous category of natural sounds. The absence of manipulation checks for perceived pitch, naturalness, pleasantness, familiarity, or induced arousal limits the ability to determine which specific auditory features drove the observed effects.

To improve transparency and reproducibility, the revised submission includes reconstructed and standardized Supplementary audio files corresponding to the four experimental conditions. These files were prepared according to the experimental criteria described in the Materials and Methods section, including auditory condition, duration, file format, sampling rate, bit depth, stereo configuration, intensity control, and absence of clipping. The Supplementary Material also provides source and license information, acoustic audit results, spectral descriptors, band-energy distribution, clipping verification, and SHA-256 hashes for traceability. Nevertheless, these files should not be interpreted as fully documented archival copies of the exact original playback files. Future research should strengthen reproducibility by archiving the exact auditory stimuli before data collection, reporting objective acoustic parameters, and including perceptual validation to confirm whether participants perceive the stimuli as intended [9,12,23].

Fourth, the study did not measure potential individual-level confounders related to participants’ pre-existing music or sound preferences. Although the auditory exposure was standardized across participants, responses to soundscapes may vary according to prior listening habits, preference for natural versus tonal sounds, familiarity with specific acoustic profiles, musical training, auditory sensitivity, or perceived pleasantness of the stimuli. These factors may have influenced emotional valence, arousal, and liking independently of the intended auditory manipulation. Future research should therefore include pre-experimental measures of music and sound preferences, perceived familiarity and pleasantness of the auditory stimuli, and relevant auditory background variables in order to determine whether sonic seasoning effects are moderated by individual differences in sound perception and preference.

Fifth, participants were not explicitly asked whether they believed the chocolate samples were identical or different across trials. Therefore, the study cannot determine whether some participants inferred the sameness of the samples, which may have influenced their responses. Future studies should include a post-experimental suspicion check or manipulation-awareness question to assess whether participants detected that the chocolate stimulus was constant across auditory conditions.

Sixth, the exclusive reliance on self-report measures—namely Visual Analogue Scales (VAS) and the Self-Assessment Manikin (SAM)—may introduce common-method bias and depend on participants’ subjective interpretation of sensory and emotional states. Although these instruments are widely validated and commonly used in sensory and affective research, they may not fully capture certain dimensions of emotional response, particularly arousal. The absence of objective or quasi-objective indicators limits triangulation of the findings. Future research should complement self-report measures with physiological and behavioral data, such as skin conductance, heart rate variability, facial expression analysis, or other non-invasive techniques, to provide a more comprehensive assessment of emotional and sensory responses during food tasting.

Seventh, although counterbalancing, palate cleansing, and waiting intervals were implemented to reduce order and carryover effects, residual sensory interference cannot be ruled out entirely in a repeated-measures tasting design. Repeated exposure to the same product across consecutive trials may have induced short-term adaptation or contrast effects, particularly for attributes such as sweetness and bitterness that are sensitive to sensory fatigue. Future research could address this limitation by incorporating longer washout periods and complementary between-subjects or mixed designs to further isolate auditory effects from residual sensory adaptation.

Eighth, the study focused on perceptual, affective, and hedonic outcomes, but did not include downstream behavioral variables such as purchase intention, willingness to pay, or choice behavior. Although liking and emotional responses are relevant proximal indicators of consumer experience, they do not, by themselves, demonstrate whether auditory modulation translates into tangible consumer decisions. Future research should therefore incorporate behavioral outcome measures to assess the applied relevance of sound-based interventions in marketing and gastronomic settings.

Ninth, although the mediation results provide evidence of an affective pathway, mediation analysis in repeated-measures designs requires careful specification and transparent handling of within-subject dependencies. In the present study, the analysis was conducted using bootstrapping procedures and within-participant contrasts; however, the absence of explicit modeling of subject-level clustering, as well as the lack of model fit indices and alternative model comparisons, limits the robustness of the conclusions. Future research should therefore consider multilevel modeling approaches or multilevel structural equation modeling, report model assumptions more explicitly, and test alternative mediation structures to better distinguish emotional processes from mechanisms such as crossmodal congruency, attentional focus, or context-induced expectations [9,11,33].

Beyond chocolate, these findings may have broader applicability to other product categories in which sonic seasoning has been examined, such as coffee and wine. Future research could explore whether similar crossmodal and affective mechanisms operate across different food and beverage contexts, thereby assessing the extent to which auditory modulation generalizes and informing multisensory design strategies.

## 5. Conclusions

This study demonstrates that auditory environment is a relevant contextual factor in chocolate tasting, influencing taste perception, emotional response, and hedonic liking in an integrated manner. The findings support a multisensory perspective in which chocolate evaluation is shaped not only by gustatory inputs, but also by auditory cues that affect both perceived taste attributes and affective responses.

A central contribution of the study is the identification of emotional valence as a relevant affective pathway linking auditory context to liking. The natural soundscape increased emotional valence and liking while reducing arousal, suggesting that positive affective quality may be more important than activation intensity in shaping favorable responses during chocolate tasting. In contrast, although the relatively high-pitched condition increased arousal and perceived acidity, this activation did not translate into higher liking.

At the same time, the persistence of direct effects of auditory context on liking suggests that emotional valence does not fully explain the observed effects. Crossmodal correspondences, attentional modulation, and context-induced expectations may also contribute to the ways in which sound shapes the overall tasting experience. Thus, the findings support a dual-route interpretation in which auditory environments influence chocolate evaluation through both taste-related crossmodal effects and affective mechanisms.

From an applied perspective, the results suggest that sound can be considered a design variable in chocolate-tasting experiences. Natural soundscapes may be especially suitable for experiences aimed at promoting calmness, positive affect, and hedonic appreciation, whereas more activating auditory profiles may be used selectively when the goal is to emphasize sharper sensory notes such as acidity. However, these applications should be interpreted cautiously because the study did not directly assess purchase behavior, willingness to pay, perceived authenticity, or origin-related meanings.

Overall, this study contributes to sonic seasoning and multisensory food research by integrating taste-intensity perception, emotional valence, arousal, hedonic liking, and mediation analysis within a single controlled within-subjects design. It also provides evidence from a chocolate-tasting context that remains comparatively underrepresented in this research stream. Future studies should extend these findings in more naturalistic tasting contexts, use more diverse consumer samples, provide acoustically validated and publicly accessible sound stimuli, incorporate individual-level moderators, and examine whether auditory environments influence behavioral outcomes and perceptions of origin, terroir, or place-based identity.

## Figures and Tables

**Table 1:** Latin-square counterbalancing (4 × 4) for auditory conditions.

Sequence/ Participant	Condition 1	Condition 2	Condition 3	Condition 4
Group 1	Relatively low-pitched	Relatively high-pitched	Nature soundscape	Silence
Group 2	Relatively high-pitched	Nature soundscape	Silence	Relatively low-pitched
Group 3	Nature soundscape	Silence	Relatively low-pitched	Relatively high-pitched
Group 4	Silence	Relatively low-pitched	Relatively high-pitched	Nature soundscape

**Table 2:** Outcomes as a function of auditory condition (Mean ± SD).

Auditory Condition	Perceived Sensory Intensity of Chocolate (VAS)			Hedonic Liking	Emotional Response During Tasting (SAM)	
	Bitterness	Sweetness	Acidity		Valence	Arousal
Relatively low-pitched	62.58 ± 9.96	43.00 ± 9.49	37.95 ± 9.73	5.78 ± 1.41	5.28 ± 1.17	5.22 ± 1.06
Relatively high-pitched	58.39 ± 10.30	43.63 ± 9.15	43.13 ± 8.63	5.46 ± 1.27	5.15 ± 1.24	5.94 ± 1.29
Nature soundscape	54.99 ± 9.59	45.45 ± 10.00	35.69 ± 9.32	6.57 ± 1.29	6.11 ± 1.19	4.44 ± 1.07
Silence	56.67 ± 10.28	45.74 ± 10.52	35.45 ± 8.05	5.99 ± 1.33	5.38 ± 1.15	5.07 ± 1.13

Note. Bitterness, sweetness, and acidity refer to perceived taste-intensity ratings measured using 0–100 visual analog scales (0 = absence of the attribute; 100 = maximum imaginable intensity). Liking was assessed on a 9-point hedonic scale. Valence reflects the pleasure–displeasure dimension, while arousal reflects activation versus calm during tasting.

**Table 3:** Repeated-measures ANOVA for the effect of auditory environment on sensory perception, liking, and emotion.

Dependent Variable	df_1_ (GG)	df_2_ (GG)	εGG	MSE	F	*p*	Partial η^2^
Bitterness (VAS)	2.81	165.61	0.936	40.17	16.95	<0.001	0.223
Sweetness (VAS)	2.64	155.51	0.879	39.49	3.13	<0.05	0.050
Acidity (VAS)	2.86	168.79	0.954	35.03	22.85	<0.001	0.279
Hedonic liking	2.75	162.50	0.918	0.59	23.85	<0.001	0.288
Emotional valence (SAM)	2.78	164.04	0.927	0.57	21.01	<0.001	0.263
Emotional arousal (SAM)	2.74	161.76	0.914	0.51	49.19	<0.001	0.455

**Table 4:** Planned contrasts for the effect of auditory environment on sensory, hedonic, and emotional outcomes.

Hypothesis	Dependent Variable	Contrast	Δ (Estimate)	95% CI	*p*
H2a	Hedonic liking	Nature soundscape vs. mean of other conditions	0.824	[0.623, 1.026]	<0.001
H2a	Emotional valence (SAM)	Nature soundscape vs. mean of other conditions	0.839	[0.651, 1.028]	<0.001
H2a	Emotional arousal (SAM)	Nature soundscape vs. mean of other conditions	−0.973	[−1.150, −0.797]	<0.001
H2b	Emotional arousal (SAM)	Relatively high-pitched vs. mean of other conditions	1.031	[0.805, 1.257]	<0.001
H2b	Perceived acidity (VAS)	Relatively high-pitched vs. mean of other conditions	6.769	[5.119, 8.420]	<0.001

Note. Contrasts are a priori planned comparisons based on adjusted marginal means from the repeated-measures ANOVA model. Ninety-five percent confidence intervals were computed from the reported standard errors (df = 59). Δ represents the estimated difference between the focal condition and the mean of the remaining sound conditions. Significance level: α = 0.05 (*p*-values adjusted for multiplicity as specified in Methods).

**Table 5:** Mediation model of the effect of auditory environment on hedonic liking through emotional response.

Effect	Contrast (X)	Path/Relationship	β (Std.)	95% CI (B) *	*p*
Path a	Nature soundscape vs. silence	X → Emotional valence	0.2537	[0.2888, 1.1408]	<0.001
Path b	—	Emotional valence → Liking	0.1634	[0.0472, 0.3188]	0.009
Indirect (a × b)	Nature soundscape	X → Valence → Liking	0.0415	[0.0257, 0.2791]	0.045
Direct (c′)	Nature soundscape	X → Liking	0.1658	[0.0306, 1.0192]	0.036
Total (c)	Nature soundscape	X → Liking	0.1825	[0.1103, 1.0455]	0.015
Direct (c′)	Relatively high-pitched vs. silence	X → Liking	−0.1860	[−1.0564, −0.1271]	0.013
Total (c)	Relatively high-pitched	X → Liking	−0.1657	[−0.9826, −0.0716]	0.026

Note. Standardized coefficients (β) are reported to facilitate comparison of effect magnitudes across paths. * The 95% confidence intervals correspond to the unstandardized coefficients (B) returned by the estimation procedure; therefore, β and its confidence intervals are not expressed on the same metric, and β is not expected to fall within the reported CI range. The indirect effect is interpreted as statistically significant when its 95% CI does not include zero.

## Data Availability

The original contributions presented in this study are included in the article/Supplementary Material. Further in-quiries can be directed to the corresponding author.

## Supplementary Materials

The following supporting information can be downloaded at https://www.mdpi.com/article/10.3390/foods15122142/s1, The supplementary audio package includes four 60-s WAV files corresponding to the experimental conditions: S1 relatively low-pitched musical soundscape, S2 relatively high-pitched musical soundscape, S3 natural soundscape, and S4 silence. The package also includes technical metadata, source and license information, acoustic audit results, band-energy descriptors, clipping verification, and SHA-256 hashes for traceability. Because the original audio files used during data collection were not deposited in a public repository at the time of the experiment, the supplied files are re-ported as reconstructed and standardized stimuli prepared according to the experimental criteria described in the manuscript.
