# Supplementary material for: Soundscapes as Sonic Seasoning of Chocolate: Effects on Taste Perception, Affect, and Liking
Source: Foods. 2026 Jun 13;15(12):2142. doi: 10.3390/foods15122142 (PMC13297889; doi:10.3390/foods15122142)
Supplement: Supplementary file 1 [file foods-15-02142-s001.zip › Auditory_S1_low_pitched_soundscape_60s/S1_low_pitched_musical_soundscape_technical_report.pdf]

Technical Report

Case S1: Relatively Low-Pitched Musical Soundscape

Reconstructed and acoustically audited auditory stimulus

Final file: S1_low_pitched_musical_soundscape_60s.wav
Source: Relaxing Jazz Music (loop) - Migfus20

1. Purpose of the Report

This report documents the auditory stimulus corresponding to condition S1: low-pitched musical soundscape. The purpose is to provide a technically defensible record for responding to the reviewer, who requested that the auditory stimuli be accessible, identifiable, traceable, and acoustically auditable. This resource replaces the previously considered non-musical drone-like candidate, because the experiment is interpreted in the context of diners exposed to music during a restaurant experience.

The analysis focuses on verifying the identity of the file, duration, format, normalization, absence of clipping, spectral distribution, band-energy profile, documentary traceability, license, and contextual relevance of the stimulus.

2. Resource Identity and Traceability

Table S1. Resource identification and traceability.

Field	Value
Experimental condition	Low-pitched musical soundscape
Final reported file	S1_low_pitched_musical_soundscape_60s.wav
Type of stimulus	Reconstructed
Source	Freesound
Source ID	723287
Resource name	Relaxing Jazz Music (loop)
Author	Migfus20
URL	https://freesound.org/people/Migfus20/sounds/723287/
License	Creative Commons Attribution 4.0 (CC BY 4.0)
Required attribution	Relaxing Jazz Music (loop) by Migfus20, obtained from Freesound, licensed under Creative Commons Attribution 4.0 (CC BY 4.0).
Declared description	Relaxing jazz/background music loop. Genre declared by the source: Jazz, Relaxing. The resource is tagged as bass, bgm, drum, jazz, loop, music, and piano.
Declared tags	bass; bgm; drum; jazz; loop; music; piano

Field	Value
Genre/category	Jazz; Relaxing / Music > Multiple instruments
SHA-256 hash of the final WAV file	4a406dbbc447e8511b0d196fe29d86b514ce5e2f56ce87692ca6ff6504a2d5e9
Processing software	MATLAB R2019a, Audio Toolbox / Signal Processing Toolbox workflow
Export date	2026-05-29 10:48:10

Analysis of the source information. The resource comes from Freesound and is licensed under Creative Commons Attribution 4.0 (CC BY 4.0). Therefore, the file may be shared and adapted, provided that explicit attribution to the author is preserved. This attribution should be retained both in the supplementary material and in any public repository used for transparency and reproducibility.

3. Technical Metadata and Processing

Table S2. Technical metadata of the original and final files.

Field	Value
Declared original format	MP3
Original format detected by MATLAB	MP3
Declared original duration	161.737 s
Detected original duration	161.611 s
Original sampling rate	44,100 Hz
Original bitrate	320 kbps
Original channels	2 / stereo
Final format	WAV
Final duration	60 s
Final sampling rate	48,000 Hz
Final bit depth	24 bit
Final channels	2 channels, stereo
Final file size	17,280,044 bytes

Technical analysis. The original file was a stereo MP3 at 44.1 kHz and 320 kbps. The final file was standardized as a stereo WAV file at 48 kHz, 24 bit, and 60.000 s. This allows the final stimulus to be reported in a homogeneous format with the other auditory files, although it should be clarified that the source resource was an MP3 file.

4. Quantitative Acoustic Audit

Table S3. Acoustic audit results.

Parameter	Value	Interpretation
RMS	−23.000 dBFS	Consistent with the target normalization level of −23 dBFS.
Peak	−3.712 dBFS	There is sufficient headroom relative to 0 dBFS; no saturation is observed.
Approximate true peak	−3.705 dBTP	Remains below 0 dBTP.
Approximate integrated loudness	−20.519 LUFS	Comparable value for reporting approximate perceptual loudness.
Crest factor	19.288 dB	Indicates the presence of moderate musical transients.
Clipping	No	Compliant: no clipping was detected.
Dominant frequency	329.590 Hz	Dominant component located in the low–mid musical region.
Spectral centroid	377.568 Hz	Low spectral center of mass; supports the low-pitched classification.
Spectral bandwidth	294.365 Hz	Limited spectral dispersion, compatible with low-activation music.
Temporal RMS variability	0.436124	Reflects the dynamic variation expected in a musical piece, rather than a static tone.

5. Band-Energy Distribution

Table S4. Band-energy distribution.

Band	Relative energy	Technical interpretation
20–250 Hz	32.473%	Low-frequency band; relevant contribution.
250–500 Hz	48.128%	Dominant low–mid frequency band.
500–2000 Hz	19.174%	Complementary mid-frequency band.
2000–8000 Hz	0.130%	High-frequency energy is practically marginal.
8000–20000 Hz	0.009%	Very-high-frequency energy is almost absent.
Total 20–500 Hz	80.601%	Main concentration in low and low–mid frequencies.
Total 2–20 kHz	0.139%	Limited high-frequency energy; favorable result for a low-pitched stimulus.

Critical interpretation of the spectral band. A total of 80.601% of the energy is concentrated between 20 and 500 Hz, whereas the energy between 2 and 20 kHz is only 0.139%. This spectral distribution supports the defensibility of the final stimulus as a relatively low-pitched musical soundscape within the reconstructed set of auditory stimuli.

6. Acoustic Comparison with the High-Pitched Stimulus

Table S5. Acoustic comparison with the high-pitched stimulus.

Indicator	S1 low-pitched musical	S2 high-pitched	Interpretation
Spectral centroid	377.57 Hz	469.61 Hz	S1 shows a lower spectral centroid than S2, supporting a relative distinction between the two musical soundscapes.
Energy 20–500 Hz	80.60%	64.72%	S1 concentrates more relative energy in the low and low–mid bands than S2.
Energy 500–2000 Hz	19.17%	35.13%	S2 shifts more strongly toward the mid-frequency band than S1.
Energy 2–20 kHz	0.139%	0.011%	Both stimuli show limited high-frequency energy, but S2 remains relatively higher in spectral centroid.

This comparison does not replace perceptual validation, but it quantitatively supports the distinction between the low-pitched musical condition and the relatively higher-pitched musical condition selected for S2. The comparison should be interpreted in relative terms, not as evidence that S2 is dominated by high-frequency energy above 2 kHz.

7. Figure Analysis

7.1. Waveform

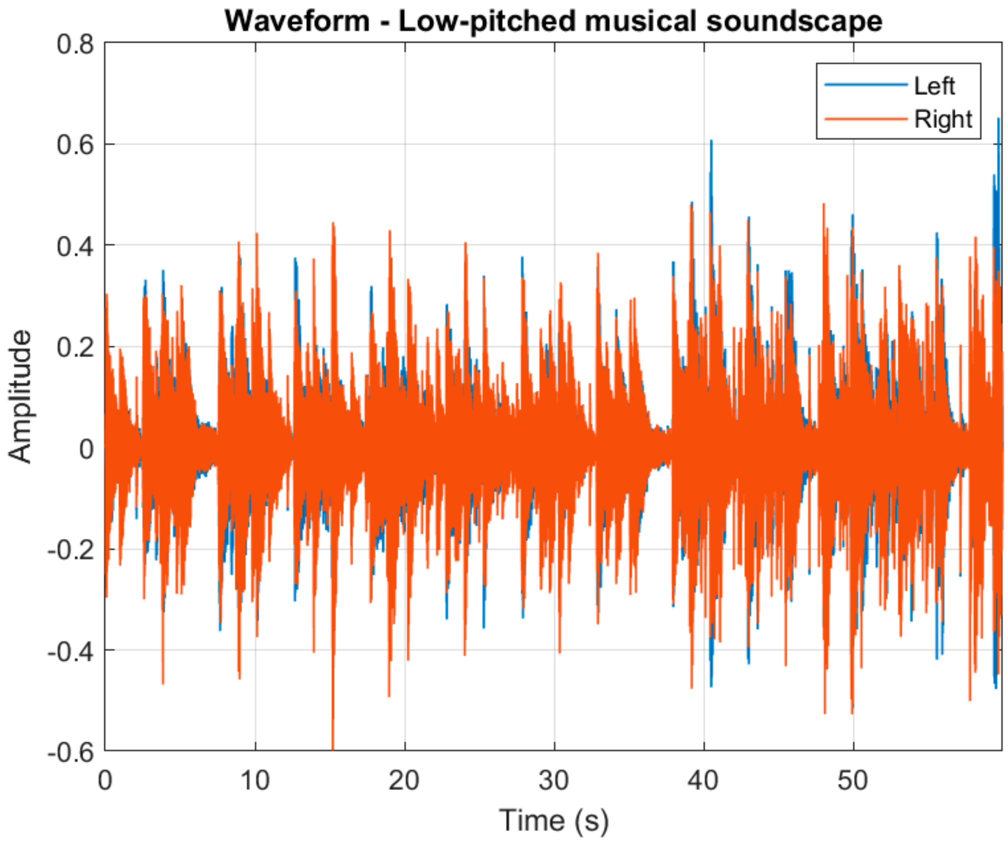

Figure S1. Waveform of the S1 low-pitched musical soundscape stimulus.

The waveform shows an active stereo signal throughout the 60-s interval, with the dynamic variation expected in a background musical piece. No fully silent segments or prolonged abrupt cuts are observed. Maximum peaks remain below digital saturation, consistent with the reported peak value of −3.712 dBFS and the absence of clipping identified in the audit. The presence of changing amplitude envelopes suggests a musical structure that is more ecologically valid for a restaurant context than the drone-like candidate initially considered.

7.2. Spectrogram

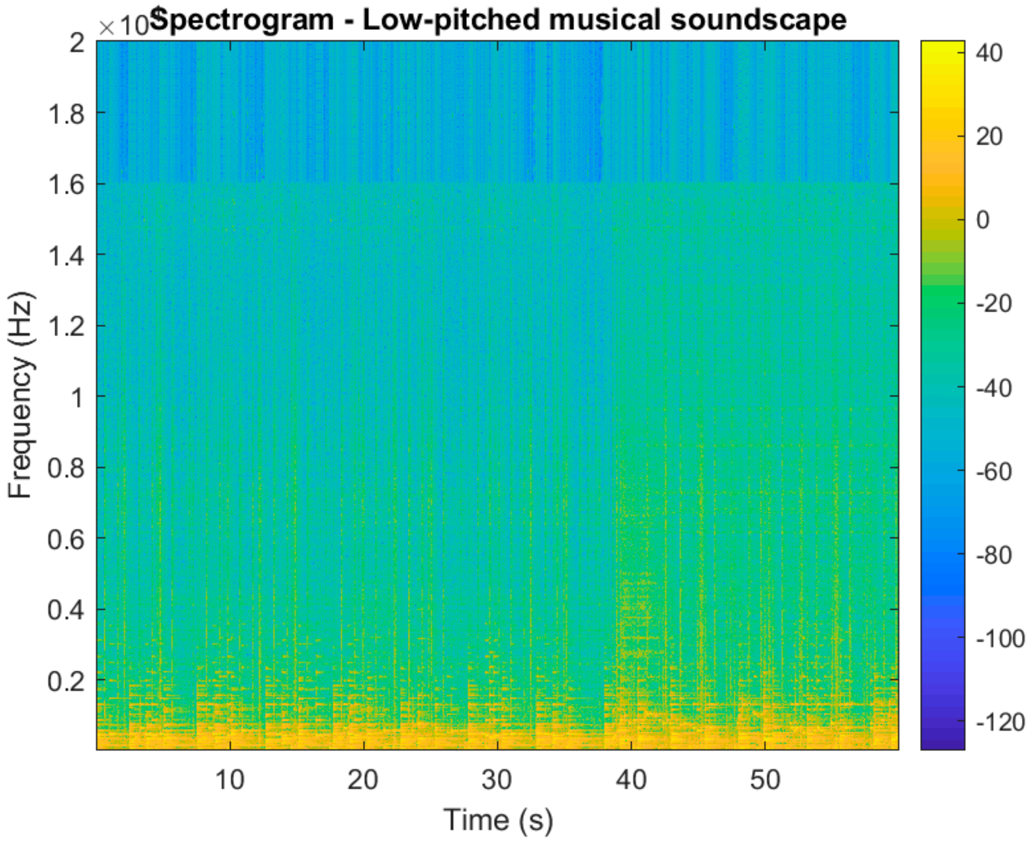

Figure S2. Spectrogram of the S1 low-pitched musical soundscape stimulus.

The spectrogram shows sustained energy mainly in the low and low–mid regions of the spectrum, with musical components distributed over time. High-frequency energy appears attenuated relative to the lower bands, which is consistent with the low percentage of energy reported between 2 and 20 kHz. The spectral texture does not correspond to a pure tone or a static drone, but rather to a musical piece with harmonic content and temporal variations, supporting its coherence with a restaurant environment.

7.3. Power Spectral Density (PSD)

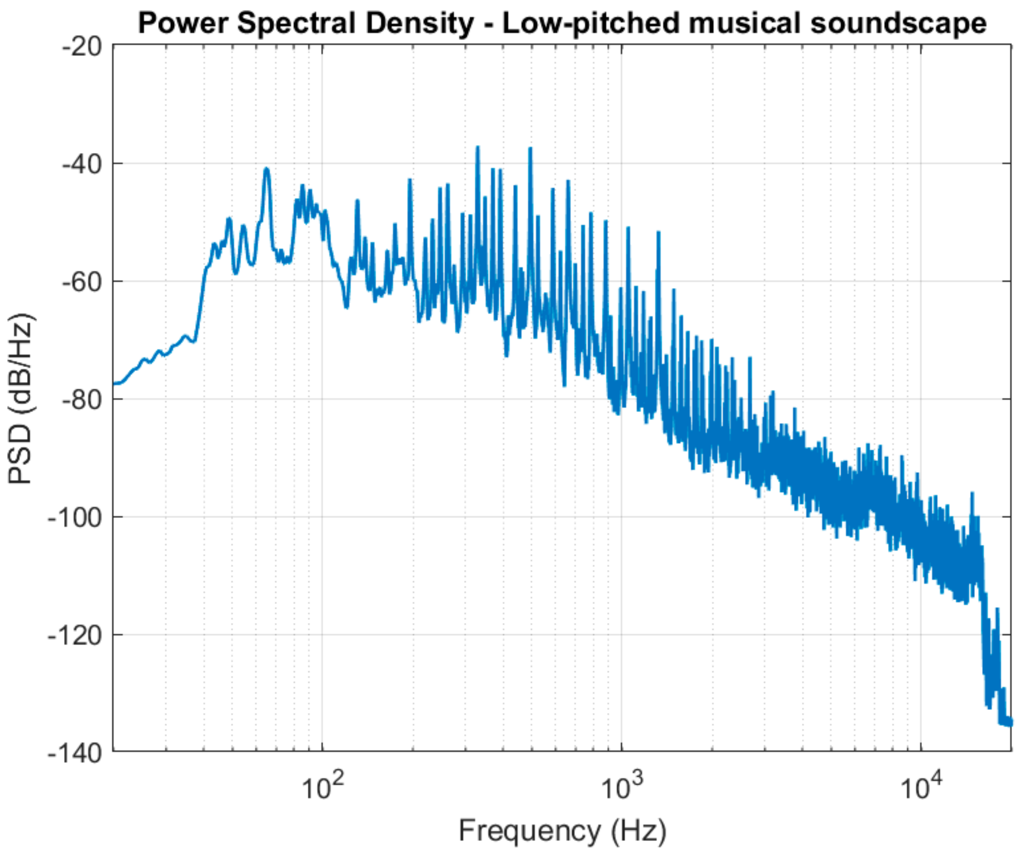

Figure S3. Power spectral density of the S1 low-pitched musical soundscape stimulus.

The PSD shows maxima and relevant components in the low and low–mid regions, approximately below 500 Hz, with a progressive decrease toward higher frequencies. This result is consistent with the estimated dominant frequency of 329.590 Hz and the spectral centroid of 377.568 Hz. Energy above 2 kHz is very limited; therefore, the file can be defended as a relatively low-pitched musical stimulus compared with the high-pitched condition.
