# Supplementary material for: Soundscapes as Sonic Seasoning of Chocolate: Effects on Taste Perception, Affect, and Liking
Source: Foods. 2026 Jun 13;15(12):2142. doi: 10.3390/foods15122142 (PMC13297889; doi:10.3390/foods15122142)
Supplement: Supplementary file 1 [file foods-15-02142-s001.zip › Auditory_S2_high_pitched_soundscape_60s/S2_high_pitched_musical_soundscape_technical_report.pdf]

Technical Report

Case S2: Relatively High-Pitched Musical Soundscape

Reconstructed and acoustically audited auditory stimulus

Final file: S2_high_pitched_musical_soundscape_60s.wav
Source: Piano loops 208 efect octave long loop 120 bpm - josefpres

1. Purpose of the Report

This report presents technical and traceable evidence for responding to the reviewer regarding the identity, license, duration, normalization, absence of clipping, and acoustic characterization of the S2 stimulus. This document does not replace the supplementary material; rather, it organizes the evidence that may be incorporated into the editorial response and the supplement.

The S2 stimulus corresponds to a high-pitched musical soundscape reconstructed from a public Freesound resource. The original file was standardized as a 60.000-s stereo WAV file at 48 kHz and 24 bit, with RMS normalization and peak control. The audit confirms the absence of clipping, exact duration, and traceability through a SHA-256 hash.

Critical observation: Although the resource is musical, piano-based, and more ecologically relevant for diners than a purely synthetic bell-like timbre, the spectral indicators show that energy is concentrated mainly below 2 kHz. Therefore, its classification as high-pitched should be defended as a relatively higher-pitched or brighter condition compared with the low-pitched musical soundscape, not as a stimulus with dominant high-frequency energy above 2 kHz.

2. Resource Identity and Traceability

Table S1. Resource identification and traceability.

Field	Reported value
Experimental condition	High-pitched musical soundscape
Final file	S2_high_pitched_musical_soundscape_60s.wav
Type of stimulus	reconstructed
Source/platform	Freesound
Source resource name	Piano loops 208 efect octave long loop 120 bpm
Author	josefpres
URL	https://freesound.org/people/josefpres/sounds/853520/
License	Creative Commons 0 (CC0)
Recommended attribution	Piano loops 208 efect octave long loop 120 bpm by josefpres, obtained from Freesound, licensed under Creative Commons 0 (CC0).
Declared category	Music > Solo instrument
Declared genre	Piano; instrumental; loop

Field	Reported value
Declared tags	120; 120bpm; free; loop; music; piano; pianomusic; reverb; samples; simple; simplesamples
Processing software	MATLAB R2019a, Audio Toolbox / Signal Processing Toolbox workflow
SHA-256 hash of the final WAV file	8c723da888ccddba740c4de6c78a612fdb8eef039cbe790dd782461c1cd8bdf4

Analysis of the source information. The resource comes from Freesound and is licensed under Creative Commons 0 (CC0), which allows its public redistribution as supplementary material. Although CC0 does not require formal attribution, crediting the author is recommended as good scientific practice. The selection is justified because it is an instrumental piano piece, which is more compatible with a restaurant context than a purely experimental bell or crystal sound.

3. Technical Metadata and Processing

Table S2. Technical metadata of the original and final files.

Parameter	Value
Declared source resource format	WAV
Declared source resource duration	132.000 s
Source sampling rate	44 100 Hz
Source bit depth	16 bit
Source channels	Stereo
Format detected by MATLAB	Uncompressed
Duration detected by MATLAB	132.000 s
Source file size	23 286 638 bytes
Final duration	60.000 s
Final sampling rate	48 000 Hz
Final bit depth	24 bit
Final channels	2
Final WAV file size	17 280 044 bytes

Technical analysis. The source file, originally a WAV file at 44.1 kHz and 16 bit, was converted into a homogeneous final WAV file at 48 kHz, 24 bit, and stereo, with an exact duration of 60.000 s. This standardization allows comparison with the other stimuli in the experimental set under the same technical format.

4. Quantitative Acoustic Audit

Table S3. Acoustic audit results.

Metric	Value	Interpretation
RMS	-23.000 dBFS	Consistent with the target RMS normalization of -23 dBFS.
Peak	-7.512 dBFS	Peak sufficiently below 0 dBFS.
Approximate true peak	-7.497 dBTP	Does not suggest problematic inter-sample overs.
Approximate integrated loudness	-20.589 LUFS	Loudness reported for comparison across stimuli.
Crest factor	15.488 dB	Indicates the difference between peak level and RMS level.
Clipping	No	Compliant: no clipping was detected.
Dominant frequency	219.727 Hz	Main dominance in the low-mid register of the piano.
Spectral centroid	469.608 Hz	Should be compared against S1 to support the relative high-pitched label.
Spectral bandwidth	287.158 Hz	Moderate spectral dispersion.
Temporal RMS variability	0.174159	Low-to-moderate dynamic variability, compatible with background music.

Normalization analysis. The final RMS practically matches -23 dBFS, while the maximum peak and true peak remain around -7.5 dB. This confirms that the stimulus was normalized conservatively, without saturation, and retains sufficient dynamic headroom for controlled playback.

5. Band-Energy Distribution

Table S4. Band-energy distribution.

Band	Relative energy	Acoustic interpretation
20-250 Hz	34.789 %	Low and low-mid band.
250-500 Hz	29.932 %	Low-mid band.
500-2000 Hz	35.129 %	Mid-frequency band, relevant for piano and harmony.
2000-8000 Hz	0.011411 %	Very low presence in high frequencies.
8000-20000 Hz	0.00002198 %	Practically no presence in very high frequencies.

Critical interpretation of the spectral band. Energy is distributed mainly between 20 and 2000 Hz, with 34.789% in 20-250 Hz, 29.932% in 250-500 Hz, and 35.129% in 500-2000 Hz. Energy above 2 kHz is almost nonexistent. For this reason, the stimulus should not be described as high-pitched on the basis of absolute dominance of high frequencies. The most defensible formulation is: high-pitched musical candidate, to be validated by comparison with the selected low-pitched musical soundscape.

6. Figure Analysis

6.1. Waveform

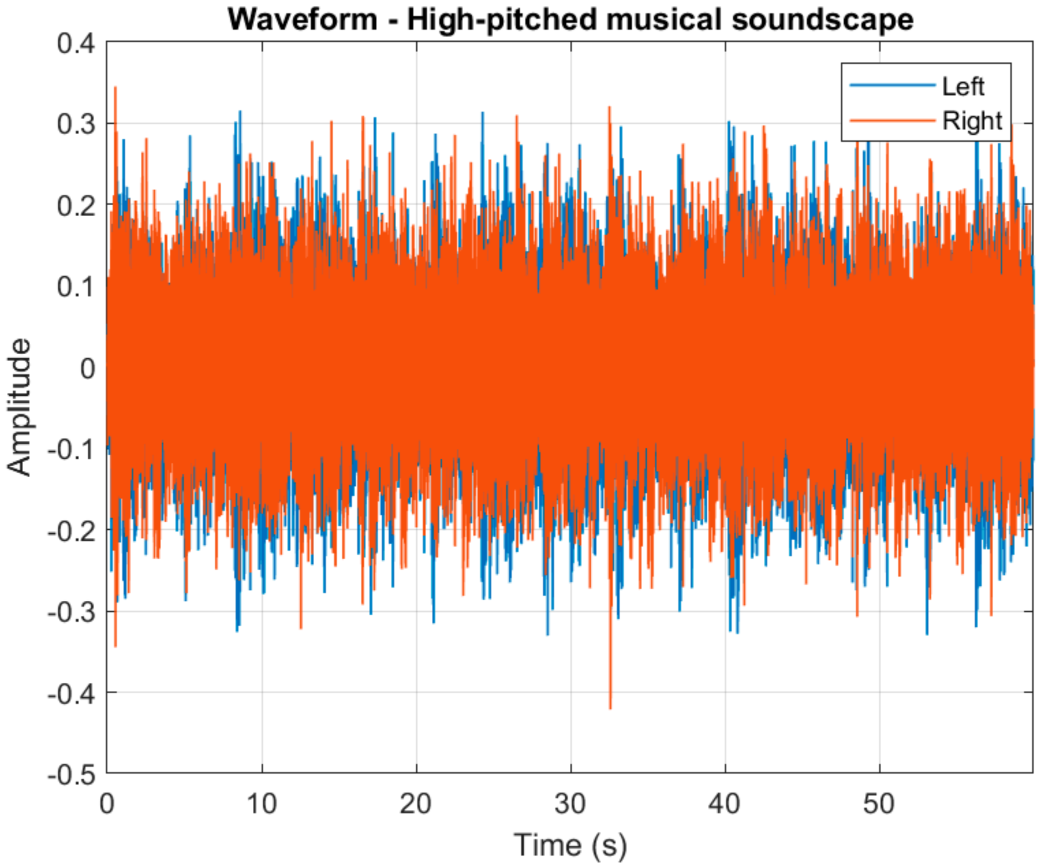

Figure S1. Waveform of the S2 stimulus: High-pitched musical soundscape.

The waveform shows a continuous stereo signal throughout the 60-s interval analyzed. No abrupt cuts, extended silent segments, or visual saturation are observed. Amplitude remains within the normalized range, and the left and right channels show similar behavior, which is consistent with a stereo musical stimulus prepared for controlled playback.

6.2. Spectrogram

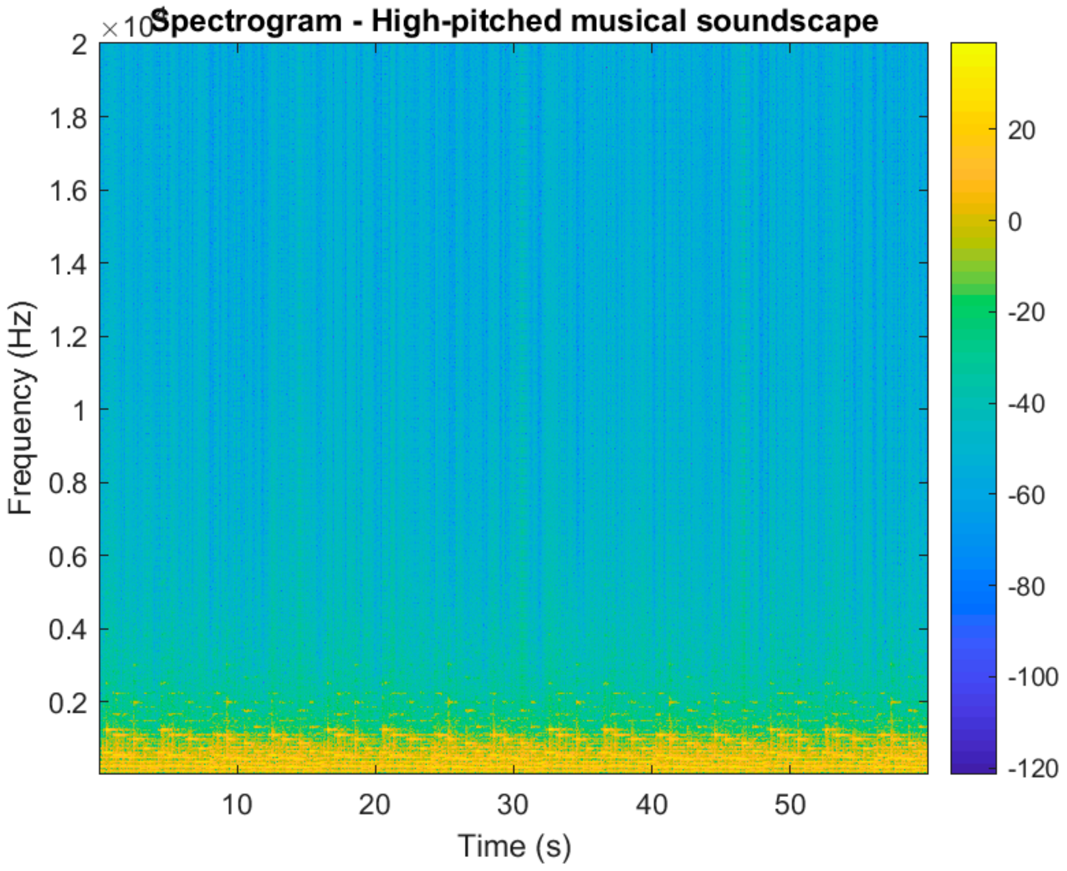

Figure S2. Spectrogram of the S2 stimulus.

The spectrogram shows a temporally stable texture and energy concentrated mainly in the low- and mid-frequency bands. The presence of content above 2 kHz is low, confirming that the high-pitched character should be understood in relative terms compared with the low-pitched musical stimulus. No severe temporal discontinuities or dominant impulsive events are observed.

6.3. Power Spectral Density

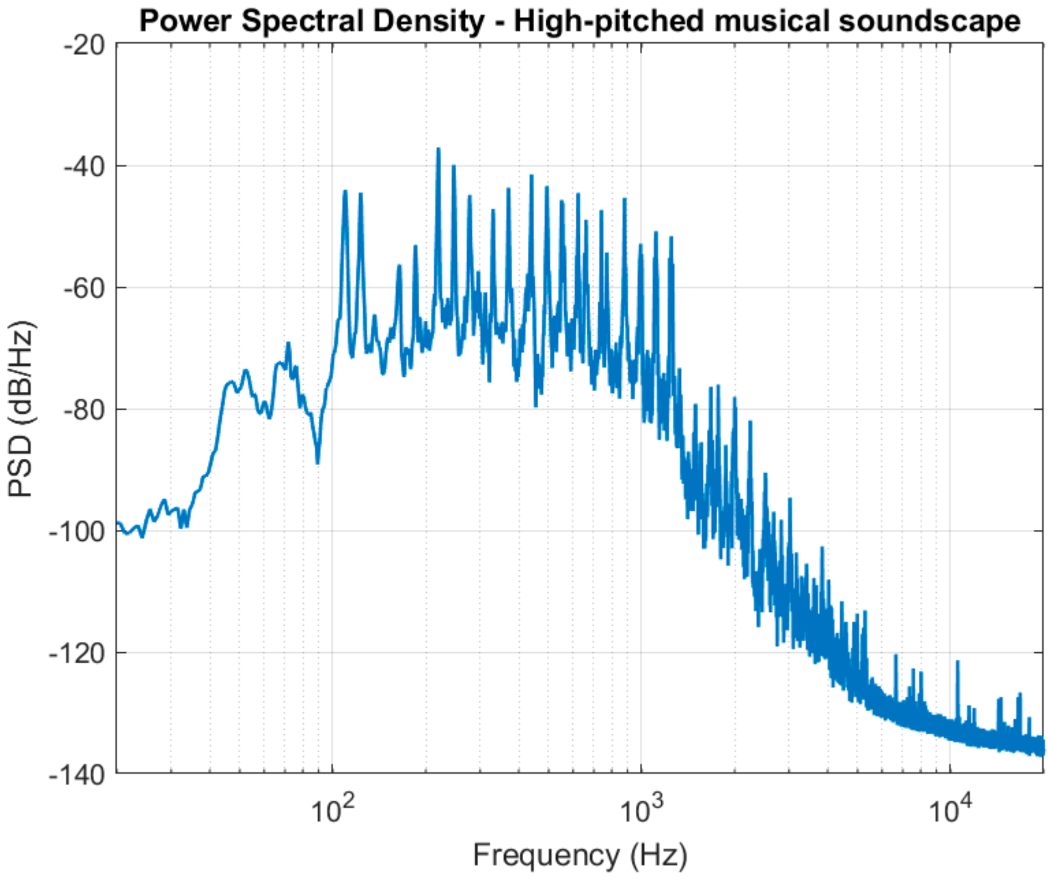

Figure S3. Power spectral density of the S2 stimulus.

The PSD shows harmonic peaks and musical content concentrated approximately between 100 Hz and 1000 Hz, with a marked decrease above 1 kHz and very low residual energy at high frequencies. The calculated dominant frequency was 219.727 Hz, and the spectral centroid was 469.608 Hz. These values are consistent with a piano in the mid-register, but not with an extremely high-pitched condition. The methodological defense should be based on comparison with the low-pitched musical stimulus and on ecological adequacy for the restaurant context.
