# Supplementary material for: Soundscapes as Sonic Seasoning of Chocolate: Effects on Taste Perception, Affect, and Liking
Source: Foods. 2026 Jun 13;15(12):2142. doi: 10.3390/foods15122142 (PMC13297889; doi:10.3390/foods15122142)
Supplement: Supplementary file 1 [file foods-15-02142-s001.zip › Auditory_S3_natural_soundscape_60s/S3_natural_soundscape_technical_report.pdf]

Technical Report

Case S3: Natural Soundscape

Reconstructed and acoustically audited auditory stimulus

Final file: S3_natural_soundscape_60s.wav
Source: Birds with Wind and Water - Echoes777

1. Purpose of the Report

This report documents the preparation, standardization, and acoustic audit of the stimulus corresponding to the Natural soundscape experimental condition. The objective is to provide a traceable response to the reviewer's observation regarding file identity, duration, technical format, intensity normalization, absence of clipping, acoustic parameters, source/license, and correspondence between the experimental description of the stimulus and the supplied file.

2. Stimulus Identity, Source, and Traceability

Table S1. Resource identification and traceability.

Field	Reported information
Experimental condition	Natural soundscape
Final file	S3_natural_soundscape_60s.wav
Type of stimulus	reconstructed
Source platform	Freesound
Source ID	849671
Source resource name	Birds with wind and water
Source file	849671__echoes777__birds-with-wind-and-water.wav
Author	Echoes777
Source URL	https://freesound.org/people/Echoes777/sounds/849671/
License	Creative Commons 0 (CC0)
Description declared by the source	Recorded in a park during a work break, featuring birds singing with wind and flowing water in the background.
Declared tags	birds; park; water; wind
SHA-256 hash of the final file	d4e7841a900bc06262bdaaa9325c2388 ecddbc4c13a9dc57d71527d2cfe74366

The source is directly and verifiably identified. The resource comes from Freesound, is associated with ID 849671, and is described as a park recording featuring birds, wind, and water in the background. This description corresponds to the Natural soundscape condition declared in the

study. The Creative Commons 0 license facilitates its public redistribution as supplementary material or as part of a data repository, provided that documentary traceability is preserved.

3. Technical Standardization of the File

Table S2. Technical metadata of the original and final files.

Parameter	Original file detected by MATLAB	Final audited file
Format	Uncompressed	WAV
Sampling rate	48000 Hz	48000 Hz
Bit depth	24 bits	24 bits
Channels	2	2
Duration	60.000 s	60.000 s
File size	17,281,588 bytes	17,280,044 bytes
Software/process	-	MATLAB R2019a, Audio Toolbox / Signal Processing Toolbox workflow
Export date	-	2026-05-28 08:07:57

The final file has a duration of 60.000 s and retains a homogeneous technical configuration of 48 kHz, 24 bit, and stereo. This standardization is consistent with the reviewer's requirement that the four stimuli be available as separate, traceable, and comparable files. The original file already had a 60-s duration and a compatible technical configuration; therefore, the processing was mainly oriented toward documenting, normalizing, and auditing the final stimulus.

4. Intensity Audit and Absence of Clipping

Table S3. Acoustic audit results.

Metric	Value	Interpretation
RMS	-24.765 dBFS	Average level documented for intensity comparison across stimuli.
Peak	-1.000 dBFS	The maximum peak is located at the intended ceiling, without reaching 0 dBFS.
Approximate true peak	-0.994 dBTP	No inter-sample peaks compromising the signal are observed.
Approximate integrated loudness	-24.328 LUFS	Approximate perceptual magnitude; complements RMS normalization.
Crest factor	23.765 dB	High peak/RMS ratio, consistent with natural transient events.
Clipping	No	No digital saturation was detected.

The RMS of -24.765 dBFS and the approximate loudness of -24.328 LUFS document the average level of the stimulus. The peak of -1.000 dBFS and the approximate true peak of -0.994 dBTP

indicate that the file was conservatively limited to avoid saturation. The crest factor of 23.765 dB is expected in a natural soundscape, where a continuous environmental background may coexist with transient events associated with birds, wind gusts, or water variations.

5. Acoustic and Spectral Characterization

Table S4. Spectral metric interpretation.

Spectral metric	Value	Interpretation
Dominant frequency	43.945 Hz	Dominant low-frequency component, compatible with an environmental wind/water background.
Average spectral centroid	265.901 Hz	Low spectral center of mass; indicates energetic predominance of low frequencies.
Spectral bandwidth	784.826 Hz	Spectral dispersion showing content broader than a pure tone.
Energy 20-250 Hz	86.545 %	Dominant band of the stimulus; associated with low natural background or continuous environmental noise.
Energy 250-500 Hz	4.102 %	Secondary contribution from low-mid frequencies.
Energy 500-2000 Hz	6.065 %	Presence of mid-frequency content compatible with environmental texture.
Energy 2000-8000 Hz	3.131 %	High-frequency components present, potentially associated with birds and environmental details.
Energy 8000-20000 Hz	0.122 %	Minor very-high-frequency content.
Temporal RMS variability	0.525623	Appreciable temporal variability, consistent with a non-stationary natural soundscape.

The spectral audit shows a strong concentration of energy in the 20-250 Hz band, with additional contributions in the mid and high bands. This does not invalidate the natural condition, because the stimulus is not intended to be tonally high- or low-pitched, but rather to represent an environment with water, wind, and birds. The dominant frequency of 43.945 Hz and the spectral centroid of 265.901 Hz suggest that the low-frequency background is energetically dominant, while the bandwidth of 784.826 Hz and the spectrogram show content distributed over time and frequency. The cumulative energy below 500 Hz reaches approximately 90.647%, whereas energy above 2 kHz reaches approximately 3.253%.

6. Analysis of Audit Figures

6.1. Waveform

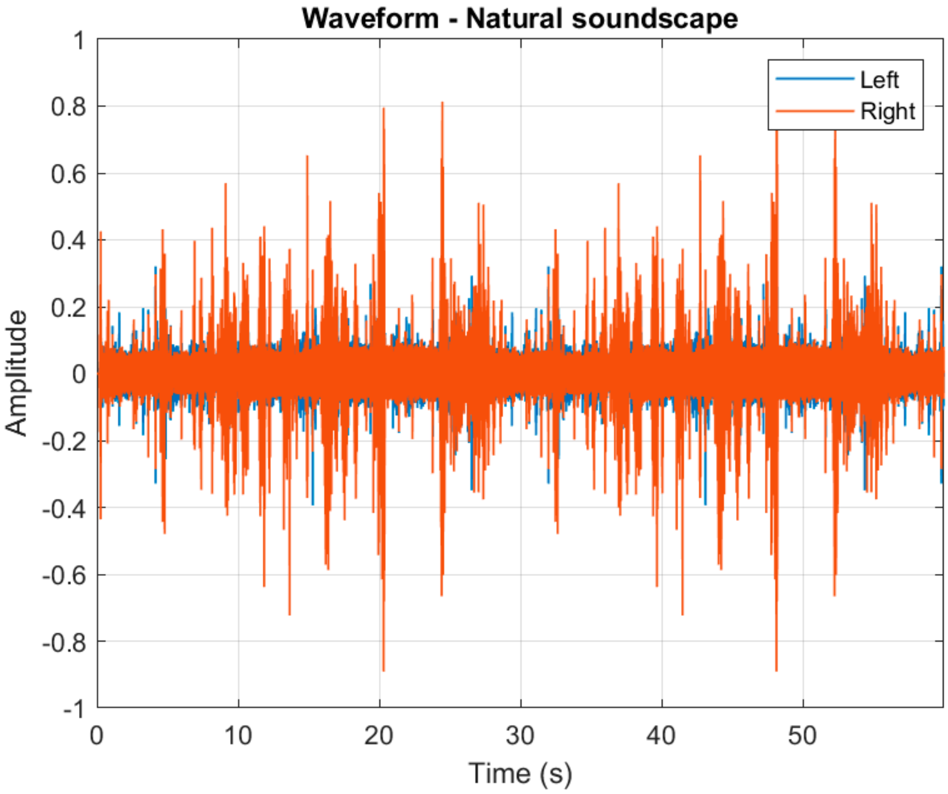

Figure S1. Waveform of the S3 stimulus: Natural soundscape.

The waveform shows a 60-s stereo signal with continuous activity and multiple transient events. Amplitude remains below digital saturation, although isolated peaks close to the -1 dBFS ceiling are observed. The temporal dynamics are consistent with a natural soundscape, where the environmental background is combined with brief higher-intensity events such as birds, wind gusts, or water variations.

6.2. Spectrogram

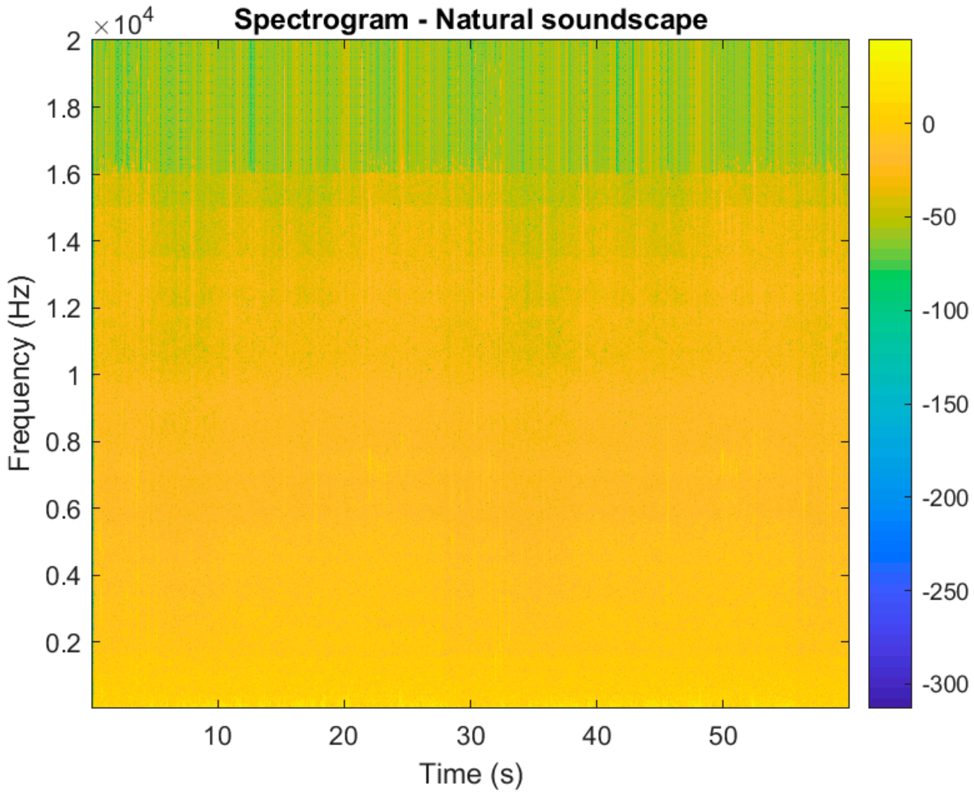

Figure S2. Spectrogram of the S3 stimulus: Natural soundscape.

Note: The inputs provided for this case included the waveform and spectrogram. The spectral parameters derived from the PSD estimation are reported in the acoustic characterization table and in the acoustic audit Excel file.

The spectrogram shows content distributed throughout the full 60-s exposure. Continuous energy is observed in the low and mid-frequency ranges, with extension toward higher regions of the spectrum. The vertical and textural variations are compatible with a non-stationary natural environment. This representation supports that the stimulus does not correspond to a dominant artificial tonal composition, but rather to a broad and changing environmental texture.
