# Supplementary material for: Soundscapes as Sonic Seasoning of Chocolate: Effects on Taste Perception, Affect, and Liking
Source: Foods. 2026 Jun 13;15(12):2142. doi: 10.3390/foods15122142 (PMC13297889; doi:10.3390/foods15122142)
Supplement: Supplementary file 1 [file foods-15-02142-s001.zip › Auditory_S4_silence_60s/S4_silence_technical_report.pdf]

Technical Report

Case S4: Silence

Reconstructed and acoustically audited auditory stimulus

Final file: S4_silence_60s.wav
Source: A minute of pure silence- Joseph Sardin

1. Purpose of the Report

This report documents the preparation, standardization, and acoustic audit of the stimulus corresponding to the Silence experimental condition. The objective is to provide a traceable response to the reviewer's observation regarding file identity, duration, technical format, absence of audible content, absence of clipping, source/license, verification hash, and correspondence between the silence condition and the supplied file.

2. Stimulus Identity, Source, and Traceability

Table S1. Resource identification and traceability.

Field	Reported information
Experimental condition	Silence
Final file	S4_silence_60s.wav
Type of stimulus	reconstructed
Source platform	BigSoundBank
Source ID	917
Source resource name	A minute of pure silence
Source file	TEST_One minute of silence (ID 0917)_BigSoundBank.com.wav
Author	Joseph SARDIN
Source URL	https://bigsoundbank.com/one-minute-of-silence-s0917.html
License	Creative Commons 0 (CC0) / Public Domain
Description declared by the source	One full minute of digital silence: no noise, no room tone, blank stereo file.
Declared tags	silence; silent; blank; test; soundscape
SHA-256 hash of the final file	730678fa62e0c96f22860fe3d79d97c7 f04a8e35ae37da036a5f5b838cd0cb7a

The stimulus is identified as the Silence condition and is associated with a specific final file that is traceable and verifiable through a SHA-256 hash. The declared source corresponds to BigSoundBank, ID 0917, described as one minute of digital silence. The CC0/Public Domain license

supports its public deposit as supplementary material or in a repository, provided that source and version traceability are preserved.

3. Technical Standardization of the File

Table S2. Technical metadata of the original and final files.

Parameter	Original file detected by MATLAB	Final audited file
Format	Uncompressed	WAV
Sampling rate	48000 Hz	48000 Hz
Bit depth	16 bits	24 bits
Channels	2	2
Duration	60.000 s	60.000 s
File size	11,521,114 bytes	17,280,044 bytes
Software/process	-	MATLAB R2019a, Audio Toolbox / Signal Processing Toolbox workflow
Export date	-	2026-05-28 08:18:38

The final file has the declared duration of 60.000 s and is standardized as a WAV file, 48 kHz, 24 bit, and stereo. The original file was detected as an uncompressed WAV file, 48 kHz, 16 bit, 2 channels, and 60 s; the processing preserves the duration and stereo configuration, but exports the final file at 24 bit to harmonize it with the other stimuli.

4. Intensity, Clipping, and Normalization Audit

Table S3. Acoustic audit results.

Indicator	Reported value / interpretation
RMS dBFS	N/A or -Inf (recorded as 65535 in Excel)
Peak dBFS	N/A or -Inf (recorded as 65535 in Excel)
Approximate true peak	N/A or -Inf (recorded as 65535 in Excel)
Approximate integrated loudness	N/A or -Inf (recorded as 65535 in Excel)
Crest factor	N/A
Clipping	No
Compliance comment	Final file exported as WAV, 60.000 s, 48 kHz, 24 bit, stereo, no RMS normalization applied to preserve digital silence, and no clipping detected if the Clipping column reports No. RMS, peak, LUFS, and spectral measures are expected to be -Inf, zero, or non-applicable for ideal silence.

The Silence condition should not be normalized to a target RMS, because doing so would introduce gain over a null signal or artificial noise. Therefore, the processing reports that no RMS normalization was applied in order to preserve digital silence. The Clipping column reports No, confirming that no saturation is present. In the RMS, peak, true peak, and LUFS fields, the Excel file displays 65535; this should be treated as an export artifact associated with non-finite or non-applicable values for ideal digital silence. For the final supplementary material, it is recommended to replace these values with “N/A” or “-Inf” and add a brief methodological note to avoid reviewer confusion.

5. Acoustic Parameters and Band-Energy Distribution

Table S4. Band-energy distribution.

Parameter	Audited value / interpretation
Dominant frequency	N/A
Spectral centroid	N/A
Spectral bandwidth	N/A
Energy 20-250 Hz	0.000 %
Energy 250-500 Hz	0.000 %
Energy 500-2000 Hz	0.000 %
Energy 2000-8000 Hz	0.000 %
Energy 8000-20000 Hz	0.000 %
Temporal RMS variability	0.000000

The spectral metrics are null or not applicable, as expected for a digital silence stimulus. The energy distribution reports 0.000% in all bands; therefore, there is no energy concentration in low, mid, high, or very high frequencies. This clearly distinguishes the Silence condition from the sound conditions S1, S2, and S3. For the response to the reviewer, it is advisable to emphasize that this condition was audited separately and that its metrics are interpreted as absence of signal, not as an acoustic stimulus with spectral content.

6. Visual Analysis of the Acoustic Figures

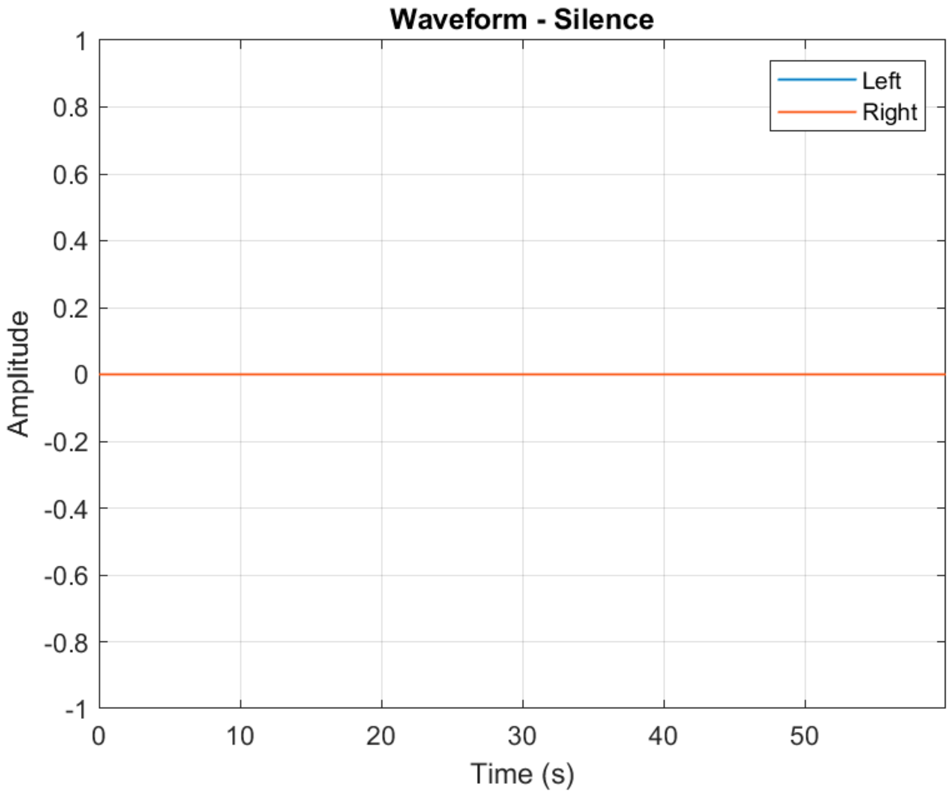

Figure S1. Waveform of the S4 stimulus: Silence.

The waveform remains on the zero-amplitude line throughout the full 60-s interval in both channels. No transients, DC offset, isolated peaks, or relevant differences between the left and right channels are observed. This figure visually confirms that the final file represents digital silence and contains no audible events introduced during processing.

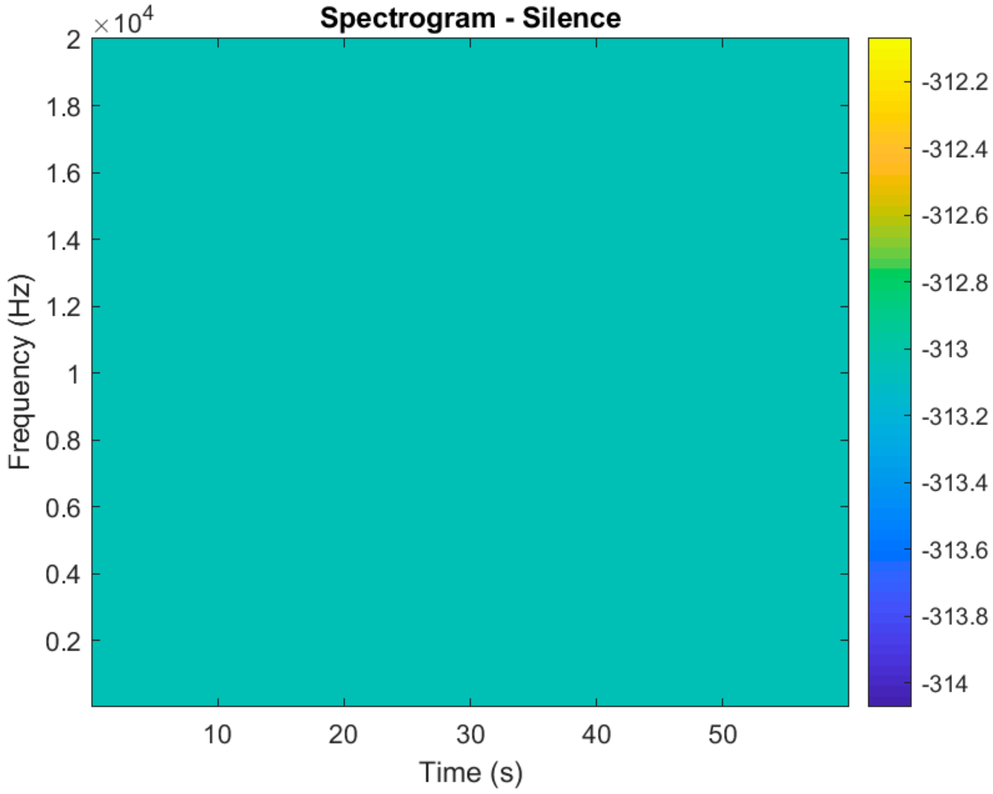

Figure S2. Spectrogram of the S4 stimulus: Silence.

The spectrogram presents a uniform field with no temporal structures or active frequency bands. The color scale is located at extremely low levels, around the numerical floor of representation. No harmonic traces, background noise, impulses, or temporal changes appear. This supports that the file has no acoustic content distributed over time or frequency.

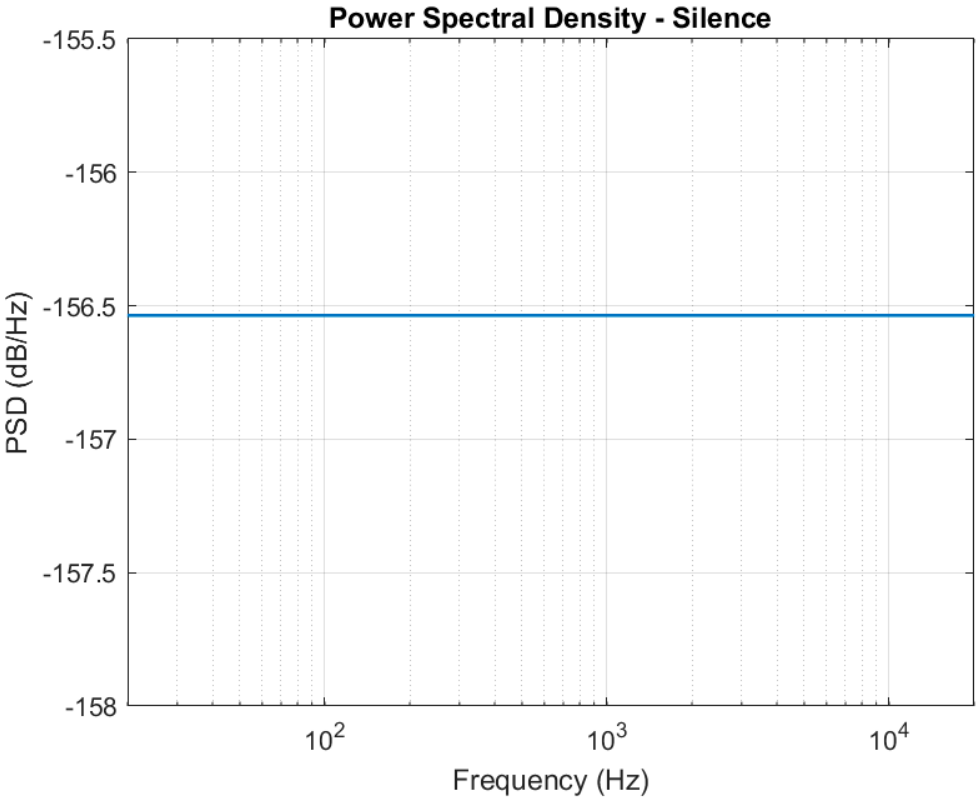

Figure S3. Power spectral density of the S4 stimulus: Silence.

The PSD appears as a flat line close to -156.5 dB/Hz. The absence of peaks, resonances, or spectral slopes confirms that there are no dominant frequency components. The represented value should be interpreted as the numerical floor of the spectral calculation and not as meaningful acoustic energy. Consequently, the PSD is consistent with a digital silence condition.
