# Supplementary figures and images for: Soundscapes as Sonic Seasoning of Chocolate: Effects on Taste Perception, Affect, and Liking
Source: Foods. 2026 Jun 13;15(12):2142. doi: 10.3390/foods15122142 (PMC13297889; doi:10.3390/foods15122142)

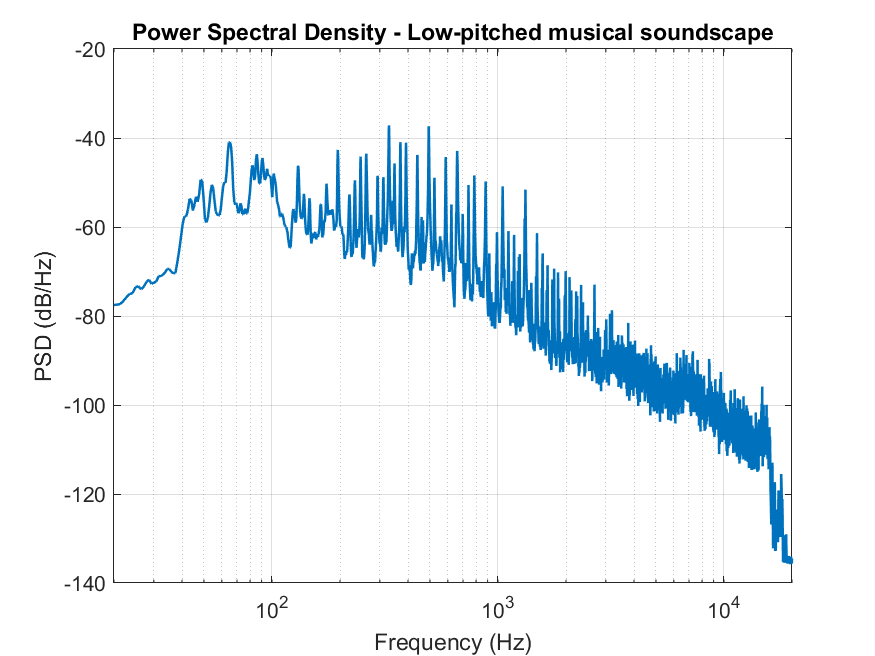

Supplement: Supplementary file 1 [file foods-15-02142-s001.zip › Auditory_S1_low_pitched_soundscape_60s/figures/S1_low_pitched_musical_soundscape_60s_psd.png]

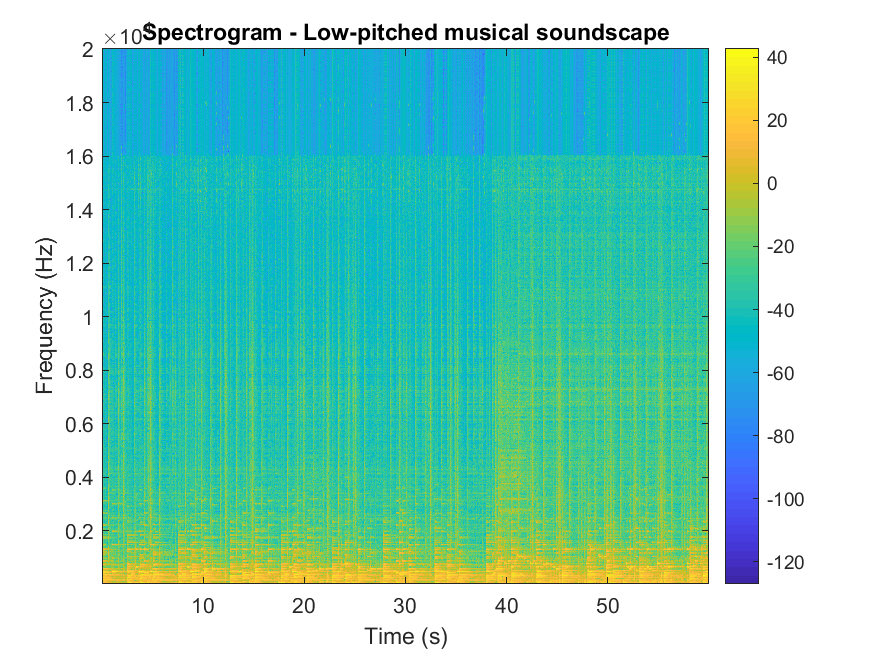

Supplement: Supplementary file 1 [file foods-15-02142-s001.zip › Auditory_S1_low_pitched_soundscape_60s/figures/S1_low_pitched_musical_soundscape_60s_spectrogram.png]

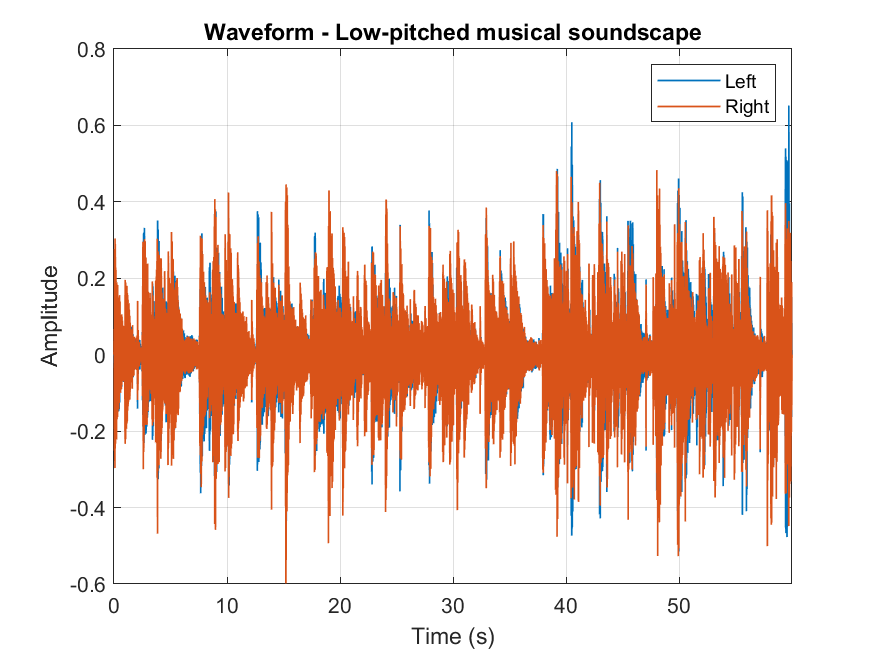

Supplement: Supplementary file 1 [file foods-15-02142-s001.zip › Auditory_S1_low_pitched_soundscape_60s/figures/S1_low_pitched_musical_soundscape_60s_waveform.png]

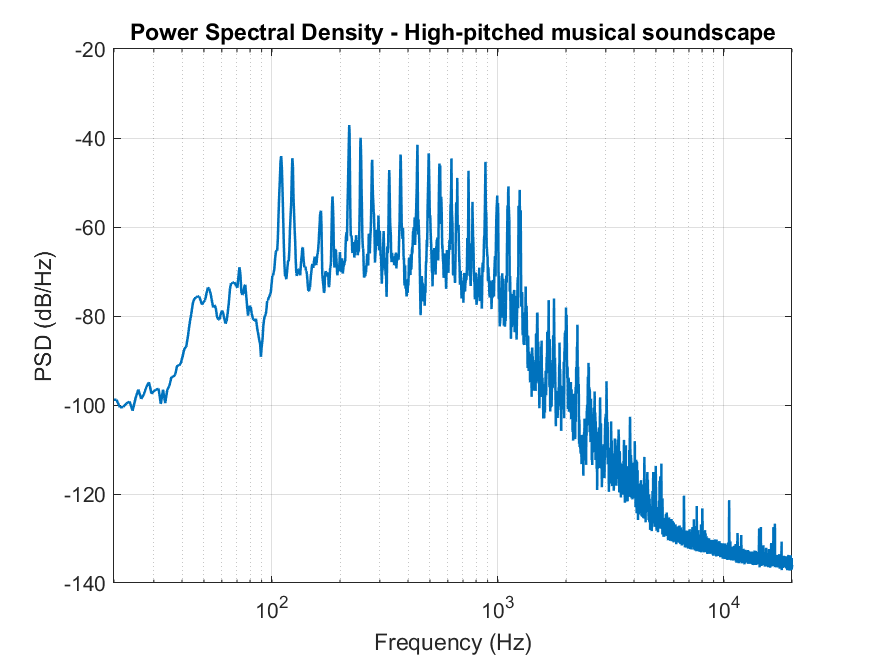

Supplement: Supplementary file 1 [file foods-15-02142-s001.zip › Auditory_S2_high_pitched_soundscape_60s/figures/S2_high_pitched_musical_soundscape_60s_psd.png]

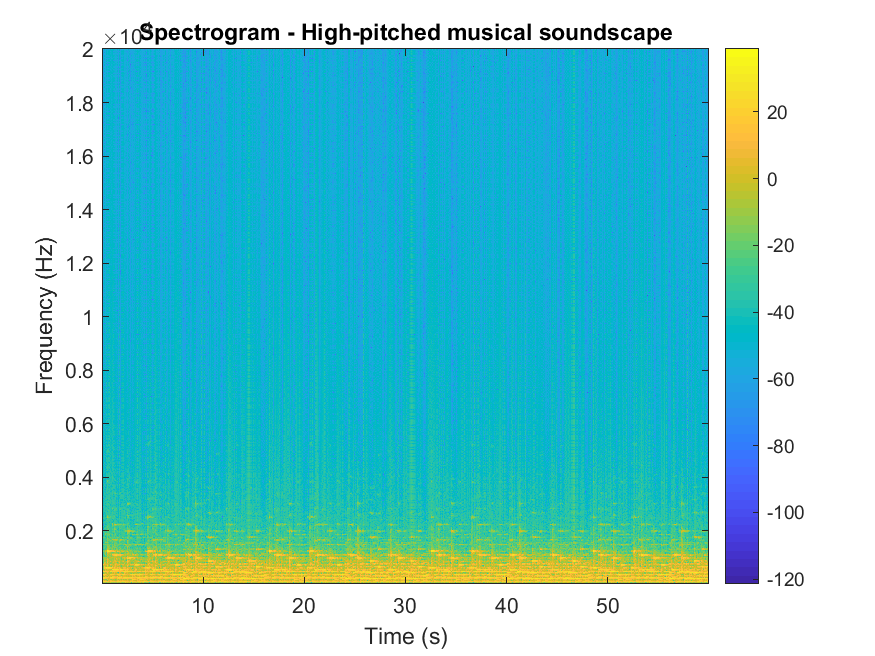

Supplement: Supplementary file 1 [file foods-15-02142-s001.zip › Auditory_S2_high_pitched_soundscape_60s/figures/S2_high_pitched_musical_soundscape_60s_spectrogram.png]

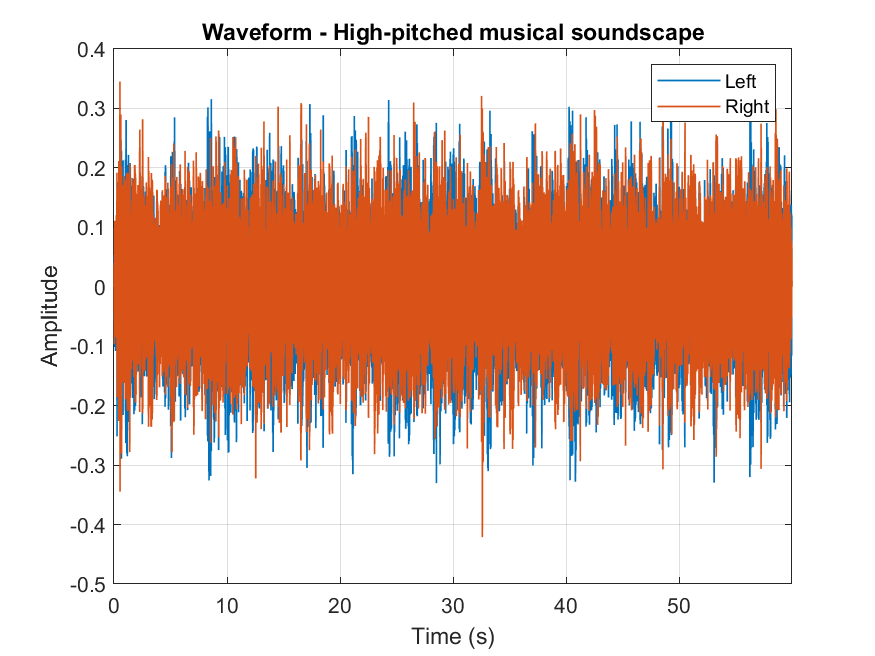

Supplement: Supplementary file 1 [file foods-15-02142-s001.zip › Auditory_S2_high_pitched_soundscape_60s/figures/S2_high_pitched_musical_soundscape_60s_waveform.png]

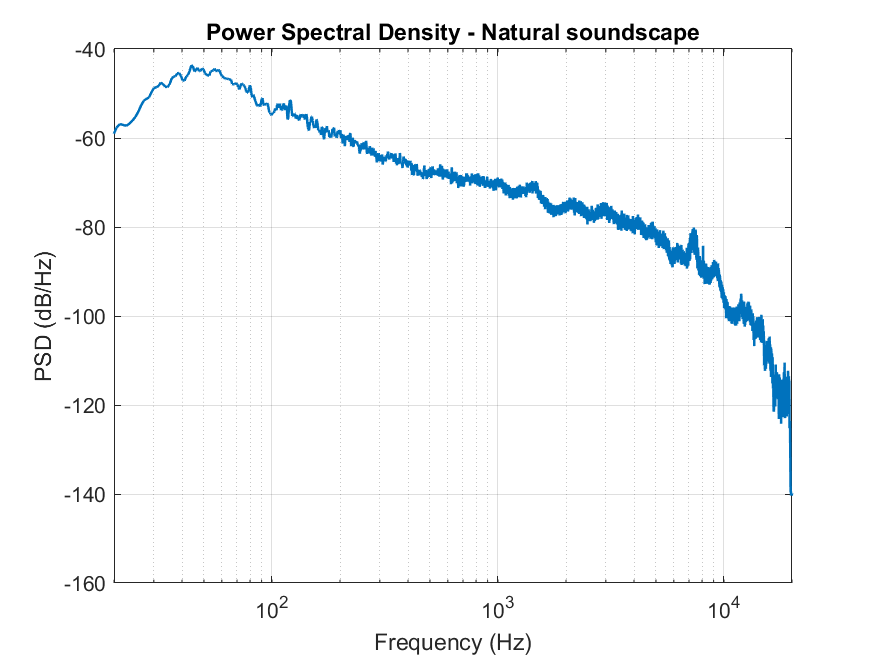

Supplement: Supplementary file 1 [file foods-15-02142-s001.zip › Auditory_S3_natural_soundscape_60s/figures/S3_natural_soundscape_60s_psd.png]

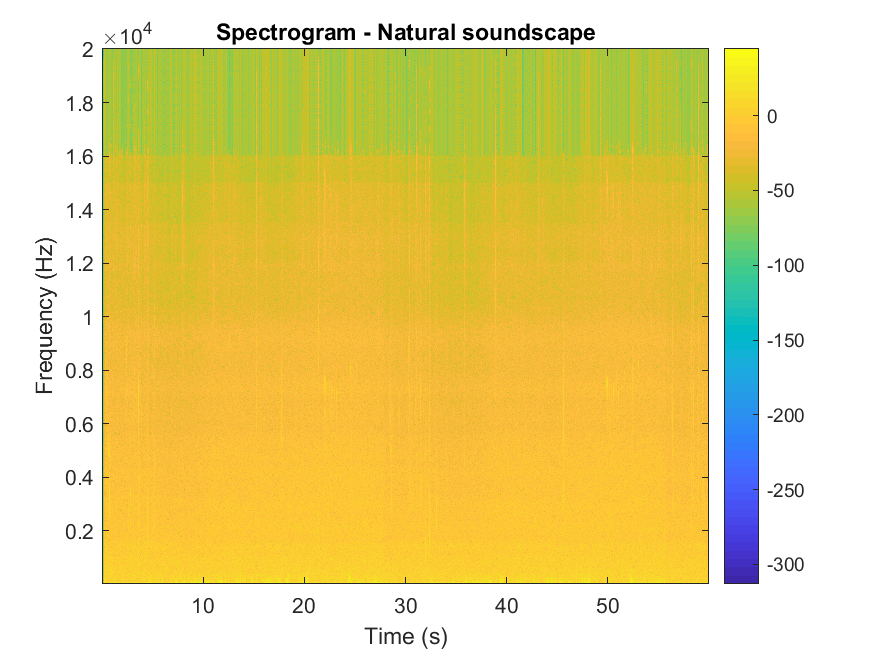

Supplement: Supplementary file 1 [file foods-15-02142-s001.zip › Auditory_S3_natural_soundscape_60s/figures/S3_natural_soundscape_60s_spectrogram.png]

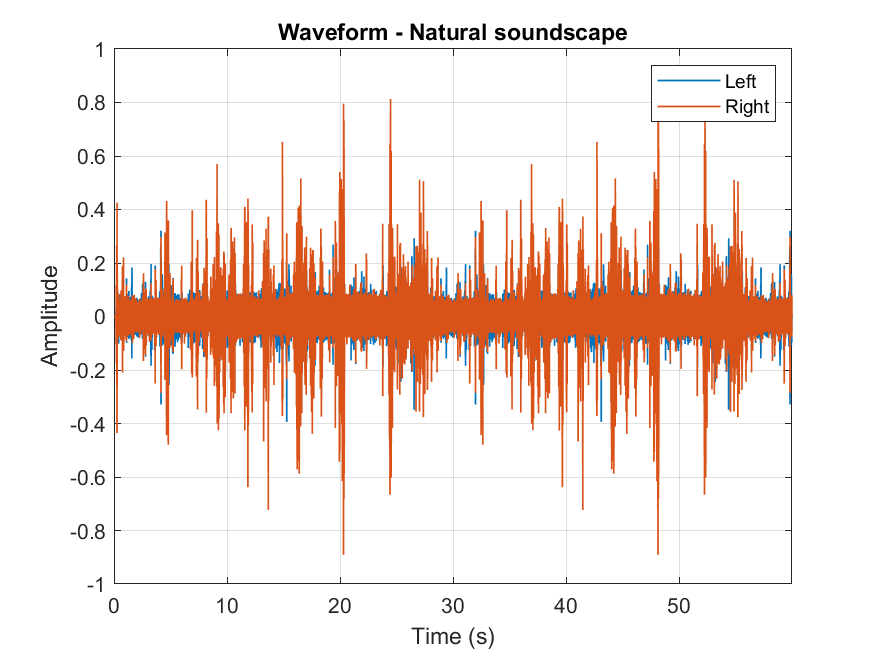

Supplement: Supplementary file 1 [file foods-15-02142-s001.zip › Auditory_S3_natural_soundscape_60s/figures/S3_natural_soundscape_60s_waveform.png]

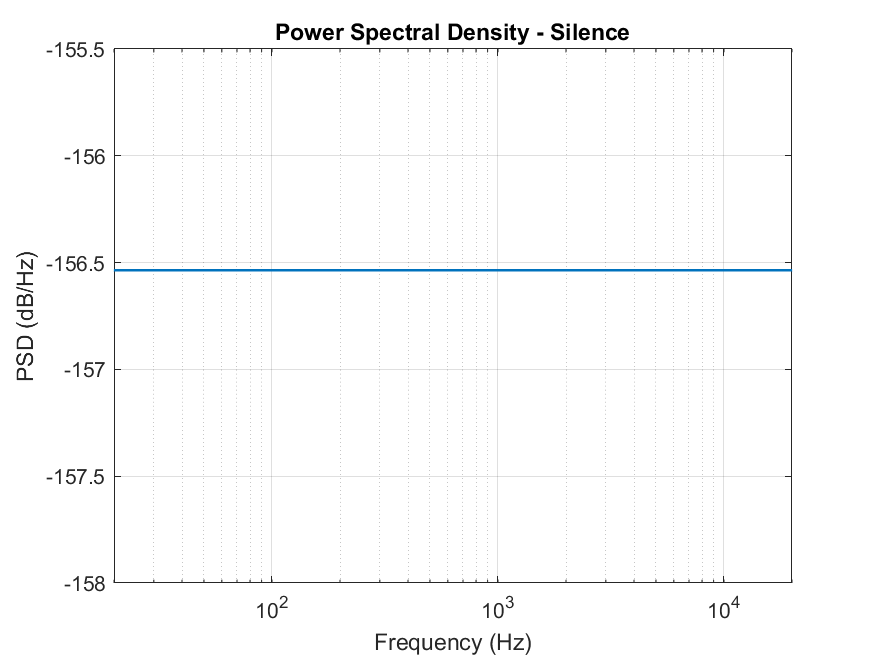

Supplement: Supplementary file 1 [file foods-15-02142-s001.zip › Auditory_S4_silence_60s/figures/S4_silence_60s_psd.png]

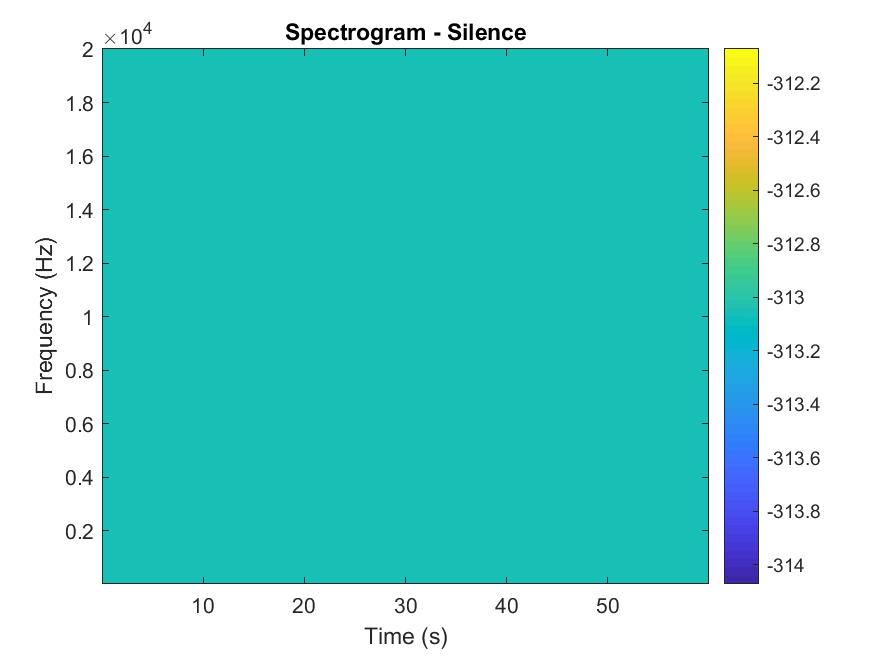

Supplement: Supplementary file 1 [file foods-15-02142-s001.zip › Auditory_S4_silence_60s/figures/S4_silence_60s_spectrogram.png]

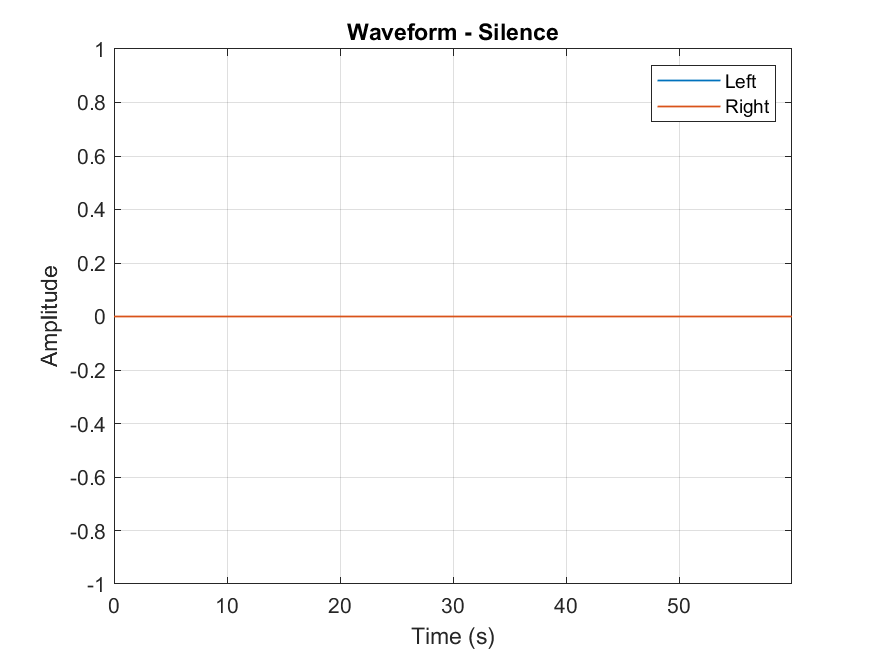

Supplement: Supplementary file 1 [file foods-15-02142-s001.zip › Auditory_S4_silence_60s/figures/S4_silence_60s_waveform.png]
